# Multi-omics and developmental comparison of direct and conventional warming methods in vitrified human and mouse cleavage-stage embryos

**DOI:** 10.1093/humrep/deag105

**Published:** 2026-07-06

**Authors:** Waner Wu, Yitong Shen, Shenghai Shen, Murong Xu, Linyao Zhang, Ka Kei Fung, Jacqueline P W Chung, Cheng-Wei Ju, Ellis K L Fok, Li-Sheng Zhang, David Y L Chan

**Affiliations:** Assisted Reproductive Technology Unit, Department of Obstetrics and Gynaecology, The Chinese University of Hong Kong, Hong Kong SAR, China; Division of Life Science, The Hong Kong University of Science and Technology (HKUST), Hong Kong SAR, China; Division of Life Science, The Hong Kong University of Science and Technology (HKUST), Hong Kong SAR, China; Assisted Reproductive Technology Unit, Department of Obstetrics and Gynaecology, The Chinese University of Hong Kong, Hong Kong SAR, China; Assisted Reproductive Technology Unit, Department of Obstetrics and Gynaecology, The Chinese University of Hong Kong, Hong Kong SAR, China; Assisted Reproductive Technology Unit, Department of Obstetrics and Gynaecology, The Chinese University of Hong Kong, Hong Kong SAR, China; Assisted Reproductive Technology Unit, Department of Obstetrics and Gynaecology, The Chinese University of Hong Kong, Hong Kong SAR, China; Division of Biology and Biological Engineering, California Institute of Technology, Pasadena, CA, USA; School of Biomedical Sciences, Faculty of Medicine, The Chinese University of Hong Kong, Hong Kong SAR, China; Division of Life Science, The Hong Kong University of Science and Technology (HKUST), Hong Kong SAR, China; Department of Chemistry, The Hong Kong University of Science and Technology (HKUST), Hong Kong SAR, China; Assisted Reproductive Technology Unit, Department of Obstetrics and Gynaecology, The Chinese University of Hong Kong, Hong Kong SAR, China

**Keywords:** vitrification, direct warming, cryopreservation, multi-omics, cleavage-stage embryo, embryo development

## Abstract

**STUDY QUESTION:**

Does direct warming preserve embryonic developmental competence and molecular integrity as well as conventional multi-step warming in vitrified human and mouse cleavage-stage embryos?

**SUMMARY ANSWER:**

Direct warming yielded comparable developmental and molecular outcomes to conventional warming in both mouse and human cleavage-stage embryos.

**WHAT IS KNOWN ALREADY:**

Conventional embryo warming uses stepwise cryoprotectant dilution to minimize toxicity and osmotic shock. Direct warming methods have been proposed, but their impact on post-warming development and the molecular comparison remains unclear.

**STUDY DESIGN, SIZE, DURATION:**

This is a controlled experimental study conducted over 18 months. A total of 490 vitrified mouse embryos and 15 donated human embryos were tested either in direct or conventional warming group. A subset underwent embryo transfer and follow-up. Parallel transcriptomic and DNA methylation profiling was performed on mouse and donated human embryos.

**PARTICIPANTS/MATERIALS, SETTING, METHODS:**

Mouse cleavage-stage embryos (C57BL/6J) were vitrified and randomly assigned to direct (n = 265) or conventional (n = 225) warming. Post-warming survival and blastocyst formation were assessed *in vitro*. A subset (n = 211) underwent embryo transfer to evaluate implantation, live birth, and postnatal development to Day 21. For molecular analysis, pooled mouse embryos from fresh, conventional, and direct groups were analyzed by bulk RNA-seq and bisulfite sequencing. Ten vitrified human embryos (n = 5 per group) were analyzed individually by scRNA-seq and scBS-seq. All procedures were conducted under standard IVF lab conditions with ethical approval.

**MAIN RESULTS AND THE ROLE OF CHANCE:**

Direct warming in mouse achieved comparable survival (95.8% vs 93.8%), blastocyst formation (88.6% vs 88.2%), implantation (82.5% vs 83.0%), and live birth rates (69.6% vs 71.1%) to conventional warming (all *P* > 0.05). Offspring showed similar growth, developmental milestones, and organ histology. Mouse transcriptome and methylome profiles revealed minimal differences and no significant pathway enrichment. In human embryos, ion channel-related gene variability was observed but without coordinated pathway disruption. Global methylation levels remained within expected developmental ranges.

**LARGE SCALE DATA:**

N/A.

**LIMITATIONS, REASONS FOR CAUTION:**

While the mouse model enables *in vivo* validation, species-specific differences in embryo size, membrane properties, and development may limit generalizability. As all embryos were cultured under optimized conditions, caution is advised when extrapolating to diverse clinical settings.

**WIDER IMPLICATIONS OF THE FINDINGS:**

To our knowledge, this is the first study to provide multi-level evidence supporting the safety and efficacy of direct warming as an alternative to conventional multi-step embryo warming protocols. By incorporating *in vivo* reproductive outcomes, postnatal development, and molecular profiling, it strengthens current evidence on the feasibility of this approach. The absence of pathway-level disruptions in transcriptome and methylome datasets suggests that direct warming does not impair essential developmental programs. These findings may support the clinical use of simplified warming procedures, especially for cleavage-stage embryo transfer or in resource-limited settings. However, further clinical studies are warranted to confirm long-term safety in humans.

**STUDY FUNDING/COMPETING INTEREST(S):**

This work was supported by Collaborative Research Fund (CRF-C4007-24E), and Early Career Scheme (ECS-26103623) from the University Grants Committee (UGC), and Health and Medical Research Fund (HMRF 12230736) from the Hong Kong Government. The authors report no competing interests.

**TRIAL REGISTRATION NUMBER:**

N/A.

## Introduction

Frozen embryo transfer (FET) has become a routine component of ART, significantly improving post-thaw survival and cumulative live birth rates (Rienzi *et al.*, 2017; Nagy *et al.*, 2020). Within vitrification protocols, the warming step is critical, as suboptimal conditions can compromise cellular integrity and developmental competence (Jin *et al.*, 2008; Seki and Mazur, 2009; Shu *et al.*, 2009).

Conventional multi-step warming remains the gold standard, sequentially exposing embryos to decreasing concentrations of non-penetrating disaccharides (e.g. sucrose or trehalose) to minimize ice recrystallization, osmotic shock, and cryoprotectants (CPAs) toxicity (Cho *et al.*, 2002; Parmegiani *et al.*, 2020). While clinically effective, this method is time-consuming, labor-intensive, and requires multiple pipetting/transferring steps. Repeated pipetting not only increases workload but also elevate the risk of embryo loss and mechanical stress. Consequently, there is growing interest in simplified protocols that streamline embryo handling without compromising safety.

In response to these challenges, simplified approaches have been developed, including one-step, single-step, and ultra-fast warming, which are generally adapted from commercial multi-step kits by using only fewer thawing solutions containing sucrose or trehalose (summarized in Supplementary Table S1). Several randomized and observational studies have reported comparable survival and clinical pregnancy outcomes between these simplified methods and conventional warming (Parmegiani *et al.*, 2020; Jiang *et al.*, 2024; Liebermann *et al.*, 2024, 2026; Chan *et al.*, 2025; Fucci *et al.*, 2025; Lammers *et al.*, 2025; Licata *et al.*, 2025; Shioya *et al.*, 2025). However, these protocols still rely on non-permeating CPAs, and subtle differences in osmotic handling may persist.

In contrast, direct warming (DW) is conceptually distinct. Instead of using thawing kits or CPA-containing solutions, embryos are transferred directly from liquid nitrogen into standard pre-equilibrated culture medium at 37°C (Chan *et al.*, 2025). The rationale is that permeating CPAs can rapidly diffuse out upon warming, while water influx is moderated by immediate exposure to isotonic culture conditions. This reduces chemical toxicity and eliminates multiple handling steps, offering potential advantages in simplicity, cost, and reproducibility. Importantly, DW is simpler compared with one-step or ultra-fast warming, which still depends on exogenous sugars to control osmotic gradients. Despite encouraging pilot results in humans (Chan *et al.*, 2025), the broader biological safety of DW remains underexplored.

Validating the comprehensive safety of new cryopreservation protocols necessitates evaluating both molecular integrity and long-term outcomes. While traditional morphological assessments provide valuable clinical benchmarks, they are inherently limited in detecting underlying transcriptional or epigenetic alterations induced by cryopreservation stress (Bhide *et al.*, 2024). To overcome these diagnostic limitations, advanced multi-omics profiling is essential. Furthermore, beyond molecular endpoints, functional validation requires robust animal models; the mouse allows embryos to be followed postnatally to evaluate *in vivo* reproductive outcomes, organ development, and long-term safety (Fernández-Gonzalez *et al.*, 2004; Feuer *et al.*, 2014). However, species-specific differences, such as the timing of zygotic genome activation and compaction, highlight the need for cautious extrapolation to humans (Gilbert, 2000; Hanna *et al.*, 2018; Schulz and Harrison, 2019).

Therefore, this study aimed to investigate whether DW can serve as a safe and effective alternative to conventional multi-step warming. We evaluated embryonic competence and reproductive outcomes in a murine transfer model, assessed the postnatal development and organ integrity of offspring, and compared transcriptomic and methylomic profiles of mouse and donated human embryos subjected to direct versus conventional warming. This multi-layered approach provides both functional and molecular evidence to support the translational relevance of DW in ART.

## Materials and methods

### Human embryos

#### Embryo source and ethical approval

Human embryos were obtained from clinical ART cycles and included two categories of surplus materials: (i) poor-quality embryos (e.g. Grade IV) that did not meet clinical criteria for transfer or cryopreservation, and (ii) embryos of transferable quality donated by patients who had completed their treatments or had no further reproductive intention. No fresh embryos were vitrified specifically for this research. All materials were originally cryopreserved for clinical use and were donated for research with written informed consent, under approval from the Joint Chinese University of Hong Kong–New Territories East Cluster Clinical Research Ethics Committee (IRB number: PWH-CT-2023-066).

#### Vitrification and warming procedures

Donated human embryos were vitrified using a commercial kit (Irvine Scientific, CA, USA; No. 90183) following the manufacturer’s instructions.

In a preliminary feasibility test of DW, donated human cleavage-stage embryos were used, as donated embryos are often not of optimal morphological quality. For downstream DW followed by multi-omics sequencing, only embryos with relatively good morphology (Grades I–II) were selected. Conventional warming followed a stepwise protocol using temperature- and concentration-controlled thawing solutions. For DW, vitrified embryos were transferred directly into pre-equilibrated (37°C) G-TL medium (Vitrolife, Gothenburg, Sweden; No. 10145) without intermediate steps, completing the warming process in ∼3 min.

### *In vitro* culture and time-lapse monitoring

After warming, embryos were cultured in G-TL medium (Vitrolife, Gothenburg, Sweden; No. 10145) under continuous observation using the EmbryoScope time-lapse incubator (Vitrolife, Gothenburg, Sweden), under low oxygen conditions (6% CO_2_, 5% O_2_). Embryo development was monitored for up to 5 days. Blastocysts were graded according to the Gardner and Schoolcraft system (Gardner *et al.*, 2000).

### Mouse embryo experiments

#### Animal handling and ethics

C57BL/6J mice (females 6–8 weeks; males ∼10 weeks) were obtained from the Chinese University of Hong Kong (Ethics Ref. 23–185-MIS) and maintained under Specific Pathogen-Free (SPF) conditions.

#### Embryo collection, vitrification, and warming

Superovulation was using pregnant mare serum gonadotropin (BioVendor, Brno, Czech Republic; No. RP1782725000) and hCG (Sigma-Aldrich, St. Louis, MO, USA; No. C1063). Zygotes were collected at 1.0 days post-coitum (dpc), and cultured in KSOM medium (Sigma-Aldrich, St. Louis, MO, USA; No. MR-106) under mineral oil (Vitrolife, Gothenburg, Sweden; No. 10029).

Vitrification utilized a DMSO-based protocol with DAP213 solution (COSMO BIO USA, Carlsbad, CA, USA; No. KYD-013-EX-0.5 and No. KYD-012-EX). Conventional warming was performed with 0.25 M sucrose (COSMO BIO USA, Carlsbad, CA, USA; No. KYD-014-EX-2) at 37°C. For DW, embryos were rewarmed directly in pre-warmed M2 (Sigma- Aldrich, St. Louis, MO, USA; No. MR-015) medium.

#### Evaluation and transfer

A subset of embryos was imaged using the Primo Vision time-lapse system (Vitrolife, Gothenburg, Sweden). Developmental stages were assessed at 1.5 and 2.5 dpc. Morphological criteria were based on the Istanbul Consensus (Alpha Scientists in Reproductive Medicine and ESHRE Special Interest Group of Embryology, 2011). Embryo survival after warming was assessed immediately, and survival was defined as ≥50% of blastomeres remaining intact.

Blastocysts were transfer using the transcervical embryo catheter transfer (Baoding Zhengmu Biotechnology, Baoding, Hebei, China) method. Each recipient received 5–12 blastocysts. Implantation and fetal development were assessed based on the number of implantation sites at 7.5 dpc and the number of live-born pups.

#### Postnatal and outcome assessment

Neonates were assessed for external morphology, developmental milestones from postnatal days 1 to 21 (PND 1–21), and internal organ appearance. Organ weights were recorded, and representative samples were fixed for histology. Paraffin sections were stained with hematoxylin and eosin (H&E) using standard protocols.

The primary outcome included post-warming morphological survival. Secondary outcomes tracked implantation rate and postnatal viability.

### Transcriptome and methylome sequencing

#### Library preparation and sequencing

Embryos were lysed using soft buffer to separate cytoplasmic RNA and nuclear DNA.

The RNA fraction was subjected to RNA library construction using the SMART-Seq Total RNA Pico Input Kit (Takara Bio, Shiga, Japan; No. 634355). All libraries were submitted to next-generation sequencing service at Novogene (Beijing, China) using Illumina platforms, with raw data generated via NovaSeq Control Software (v1.7; Illumina, San Diego, CA, USA). Paired-end 150 bp mode was selected for all samples. Gene expression values were calculated as transcripts per million (TPM).

The DNA fraction was subjected to bisulfite library construction using the Pico Methyl-Seq Library Kit (Zymo Research, Irvine, CA, USA; No. D5456), following its standard low-input protocols. Paired-end 150 bp mode was selected for all samples. Conversion efficiency was evaluated using unmethylated lambda spike-in DNA.

#### Bioinformatics and statistical analysis

Detailed bioinformatics pipelines, including raw read quality control and preprocessing parameters, are provided in the Supplementary Materials and Methods.

Briefly, for transcriptome profiling, filtered RNA-seq reads were aligned to the reference genomes (GRCm39 or GRCh38) using HISAT2 (v2.2.1; Center for Computational Biology, Johns Hopkins University, Baltimore, MD, USA) (Kim *et al.*, 2015). Gene-level quantification was performed via featureCounts (v2.0.7; Subread package, Walter and Eliza Hall Institute of Medical Research, Melbourne, Australia), and differential expression was analyzed using DESeq2 (v1.42.0; Bioconductor Open Source Project) (Love *et al.*, 2014). Differentially expressed genes (DEGs) were defined by an absolute log_2_ fold change >1 and an adjusted *P*-value < 0.05.

For DNA methylation analysis, quality-filtered whole-genome bisulfite sequencing (WGBS) reads were aligned to bisulfite-converted genomes using Bismark (v0.24.2; Babraham Bioinformatics, Cambridge, UK) (Krueger and Andrews, 2011). Differentially methylated regions (DMRs) and genes (DMGs) were identified using methylKit (v1.34; Bioconductor Project, Seattle, Washington, DC, USA) (Akalin *et al.*, 2012), applying stringent thresholds (minimum 5× coverage, ≥20% methylation difference, and *q* < 0.01). Functional enrichment and gene set enrichment analysis (GSEA) for both DEGs and DMGs were executed using the clusterProfiler package (v4.12.0; Jinan University, Guangzhou, China) (Yu *et al.*, 2012).

### Statistical analysis

Sample size was calculated using G*Power (Version 3.1; Heinrich Heine University Düsseldorf, Düsseldorf, Germany) based on the primary outcome of morphological survival. To detect a clinically relevant absolute difference of 15%, derived from published key performance indicators for vitrified embryos (competence threshold 70% vs benchmark 85%) (Alpha Scientists in Reproductive Medicine, 2012), 199 embryos per group were required (α = 0.05, power = 0.95). To minimize allocation imbalance, embryos were alternately assigned to the respective warming groups; formal randomization and blinding were not feasible due to procedural constraints.

Data were analyzed using SPSS (v29.0.1.0; IBM Corp., Armonk, NY, USA) and GraphPad Prism (v10.0.3; GraphPad Software, Boston, MA, USA). Continuous variables were compared using Student’s *t*-test or Mann–Whitney *U*-test, and categorical variables using chi-squared test or Fisher’s exact test, as appropriate. Statistical significance was set at *P* < 0.05.

## Results

### Preliminary data in human cleavage-stage embryo

**Figure 1.**
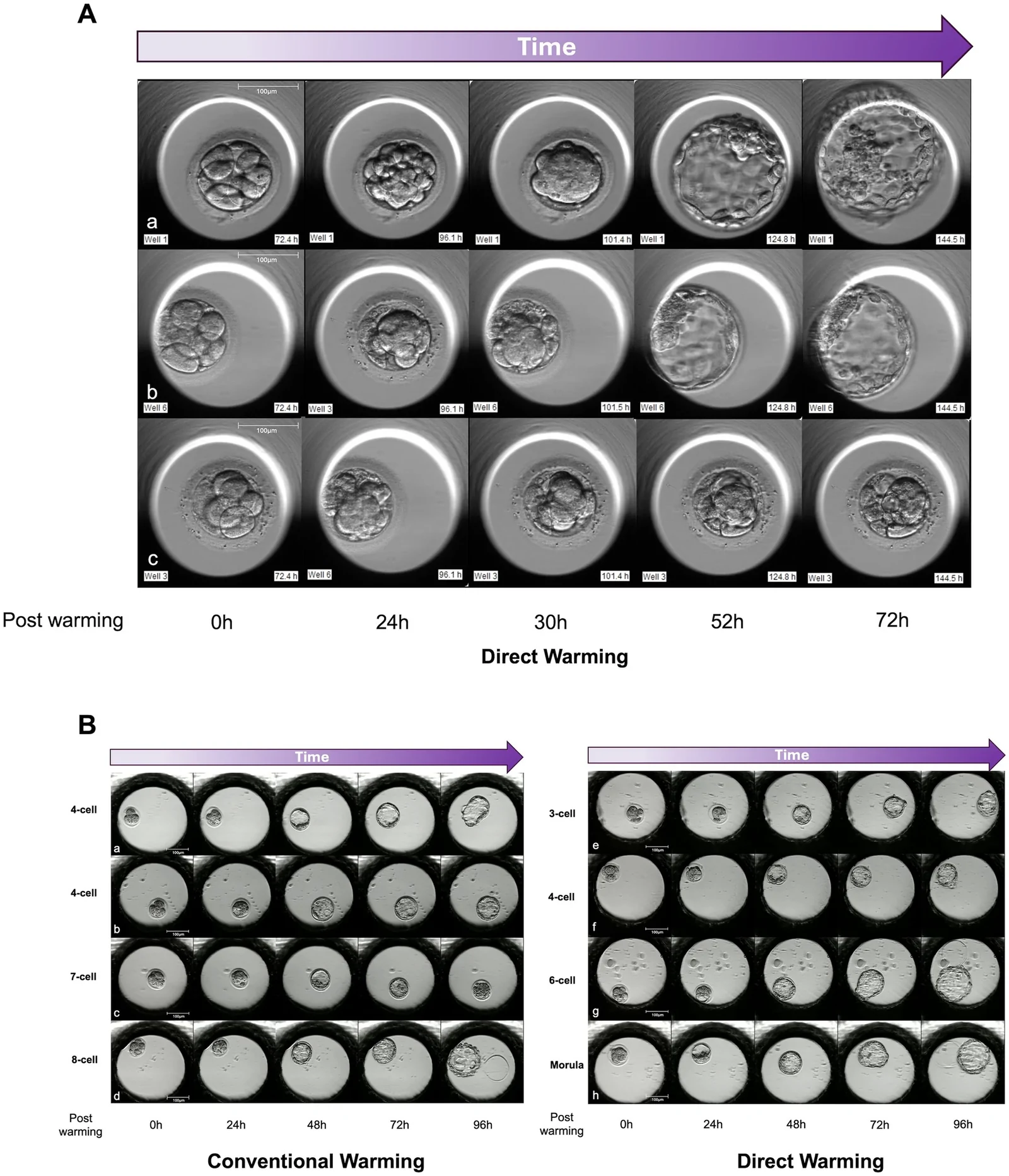
**Post-warming developmental dynamics of human and mouse cleavage-stage embryos.** (**A**) Representative time-lapse images of three human embryos (**a-c**) captured at 0, 24, 30, 52, and 72 h post-direct warming. Embryos were cultured and monitored continuously using the EmbryoScope^®^ time-lapse incubation system. Embryos a and b developed into expanded blastocysts graded as 5BB and 4BB, respectively, while embryo c arrested at the early blastocyst stage. Blastocyst grading was performed according to the Gardner and Schoolcraft system. Abbreviations and grading criteria: 4, expanded blastocyst (blastocyst cavity completely fills the embryo, zona pellucida thinning); 5, hatching blastocyst (trophectoderm has started to herniate through the zona pellucida); first ‘B’, inner cell mass (ICM) grade B (several cells, loosely grouped); second ‘B’, trophectoderm (TE) grade B (few cells forming a loose epithelium). (**B**) Representative time-lapse images of mouse cleavage-stage embryos after conventional versus direct warming. Embryos rewarmed using either the conventional multi-step method or the direct warming method were cultured under identical conditions and continuously monitored over a 96-h period using time-lapse imaging. Each row (a–h) shows a representative embryo from each group, with snapshots captured at 0h, 24h, 48h, 72h, and 96h post-warming. The labels on the left indicate the number of morphologically intact blastomeres immediately after warming, used as a surrogate marker of initial survival. Morphological changes such as cleavage progression, compaction, cavitation, and blastocyst formation are shown across timepoints. Scale bars are included in each image.

Given the clinical reality that some patients possess only a limited number of embryos with relatively low blastocyst formation rates (The Vienna Consensus, 2017), it is essential to evaluate whether DW is applicable at the cleavage stage. In this preliminary test, six donated human cleavage-stage (1× 6C grade IV, 1× 7C grade IV, 3× 8C grade IV, and 1× 8C grade II) embryos were subjected to the DW procedure. Embryos were categorized into four morphological grades (Grades I–IV) based on blastomere symmetry and fragmentation percentage, adapted from the Istanbul consensus (2011) (Alpha Scientists in Reproductive Medicine and ESHRE Special Interest Group of Embryology, 2011). In this scoring system, Grades 1–3 correspond to ‘Good’, ‘Fair’, and ‘Poor’ categories, respectively, while Grade 4 was utilized to denote very poor-quality embryos with extensive fragmentation (>35%) and cellular abnormalities. Among all poor grading embryos, four demonstrated >50% blastomere survival and resumed development post-warming. All four progressed to the blastocyst stage, and notably, two grade IV cleavage stage embryos grown into good-quality blastocysts (2× 4BB) based on standard morphological criteria. According to the Gardner grading system, 4BB represents an expanded blastocyst with a prominent, loosely grouped inner cell mass (grade B) and a cohesive layer of trophectoderm cells (grade B) (Gardner *et al.*, 2000). Representative images from the time-lapse videos are shown in Fig. 1A.

Considering that the average blastocyst formation rate for vitrified-warmed human cleavage-stage embryos in clinical practice typically ranges from ∼50% to 70% (Wang *et al.*, 2016; Le *et al.*, 2021; Shioya *et al.*, 2025), these initial results are unexpectedly encouraging. The results suggest that DW can preserve embryo viability and developmental competence even at the cleavage stage.

### Post-warming development of mouse embryos

**Figure 2.**
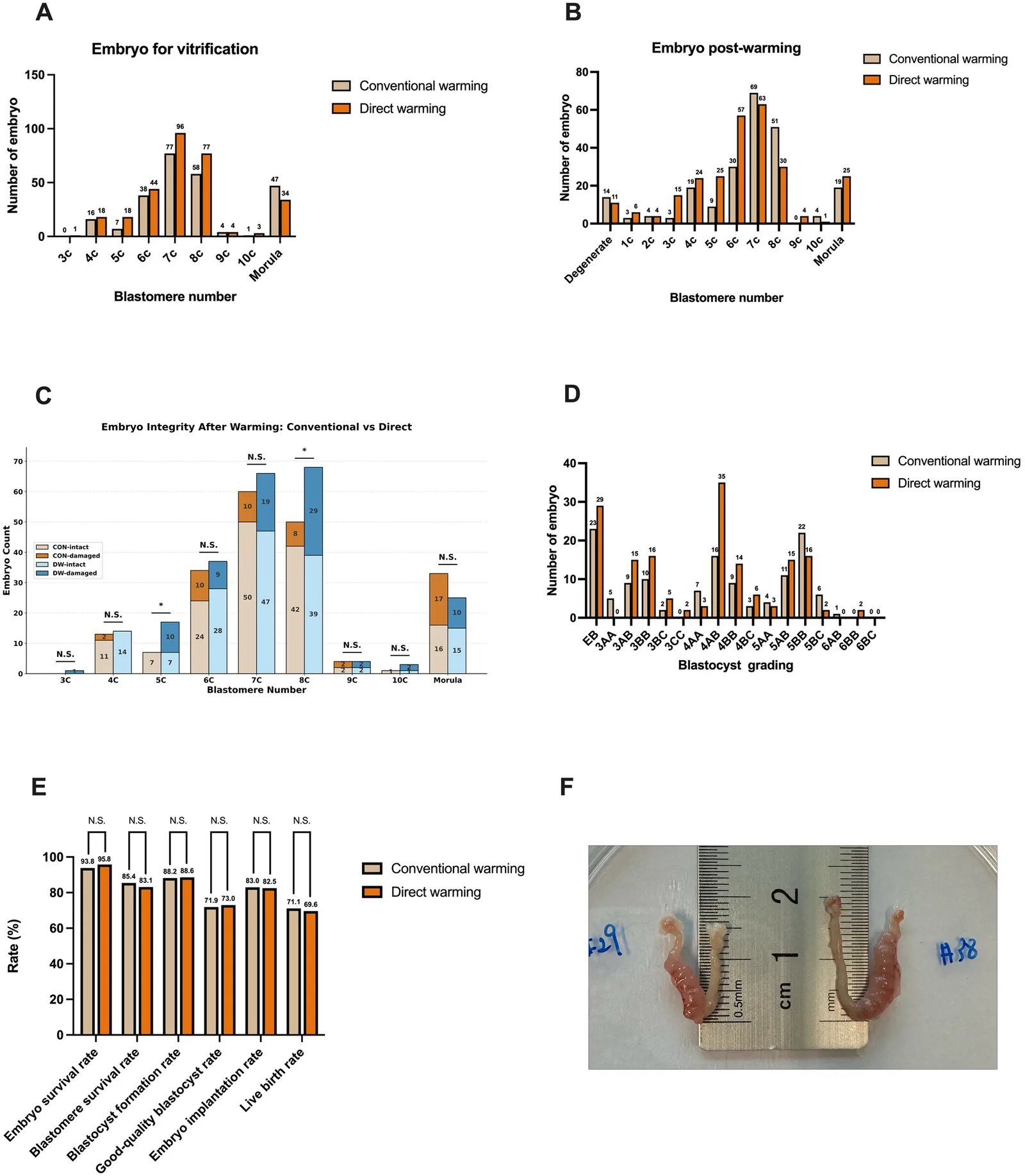
**Developmental characteristics and post-warming integrity of mouse cleavage-stage embryos following conventional or direct warming.** (**A**) Distribution of embryos by blastomere number prior to vitrification in the conventional warming (brown) and direct warming (orange) groups. (**B**) Distribution of blastomere number immediately post-warming. (**C**) Grading outcomes of embryos that developed to the blastocyst stage, based on expansion and inner cell mass/trophectoderm morphology. (**D**) Post-warming embryo integrity categorized by blastomere number. Embryos were classified as intact (all blastomeres morphologically preserved) or damaged (at least one lysed or fragmented blastomere). (**E**) Comparison of key outcome rates between groups, including embryo survival rate, blastomere intact rate, blastocyst formation rate, good-quality blastocyst rate, embryo implantation rate, and live birth rate. (**F**) Gross morphology of dissected uterine horns from pregnant mice. Representative images show the uterine horns from sample #29 (left) and sample #38 (right) containing visible implantation sites. A metric ruler is placed in the center for size reference (scale markings indicate centimeters [cm] and millimeters [mm]). Data are presented as absolute counts (A–D) or percentages (E). Statistical comparisons were performed using the chi-squared test or Fisher’s exact test, as appropriate. *P < 0.05, **P < 0.01; ns, not significant.

To assess post-warming embryo viability and developmental potential, we analyzed time-lapse videos of cleavage-stage embryos cultured after warming using either conventional or direct protocols. Representative images from these videos are shown in Fig. 1B. Most embryos in both groups successfully developed into blastocysts following warming. A total of 1960 zygotes were collected from superovulated female mice after mating. Of these, 965 embryos were selected for vitrification based on developmental stage, representing 49% of the collected zygotes. Only embryos that had reached the 6–10 cell stage (2.5dpc) or morula stage at the time of assessment were considered suitable for freezing. The average blastomere number prior to vitrification was similar between the two groups (cell number), indicating a comparable baseline developmental stage (Fig. 2A).

We compared the survival of blastomere numbers after warming between the two groups (Fig. 2B). The overall survival rates were similar, with most embryos in both groups falling within the 6- to 8-cell range (66.7% vs 56.7%, NS). Although Fig. 2C showed a lower proportion of intact embryos in the DW group at the 5-cell and 8-cell stages, this did not appear to affect overall embryo developmental capacity. The survival rate after warming was 93.8% (211/225) in the conventional group and 95.8% (254/265) in the DW group, no statistically significant was observed. When embryos were further cultured to the blastocyst stage (3.5dpc), the blastulation rate outcomes were also comparable between the two groups (88.2% vs 88.6%, NS), with no noticeable shift in quality patterns based on the Gardner grading system (Fig. 2D) (Gardner *et al.*, 2000).

*In vitro* developmental outcomes following conventional versus DW were assessed by evaluating four key parameters: embryo survival rate, blastomere survival rate, blastocyst formation rate, and the proportion of good-quality blastocysts (≥3BB). As shown in Fig. 2E, there were no statistically significant differences between the two groups across all evaluated metrics. The embryo survival rate was 93.8% in the conventional group and 95.8% in the DW group (NS), while the blastomere survival rate was 85.4% (1355/1586) versus 83.1% (1649/1985), respectively (NS). Similarly, the blastocyst formation rate was 88.2% in the conventional group compared to 88.6% in the DW group (NS). The proportion of good-quality blastocysts was also comparable between groups (71.9% vs 73.0%, NS). These results suggest that DW is not inferior to conventional multi-step warming in supporting early *in vitro* developmental competence of vitrified cleavage-stage mouse embryos.

### *In vivo* reproductive outcomes following embryo transfer

To evaluate the long-term developmental competence of embryos warmed by different methods, warmed cleavage-stage embryos were transferred into pseudopregnant female mice using the non-surgical TCET method. Each recipient received 5–12 embryos. Implantation and fetal development were assessed based on the number of implantation sites at 7.5 dpc and the number of live-born pups.

As shown in Supplementary Table S2, implantation was assessed by sacrificing recipient females at 7.5 dpc and examining the uterus for the presence of implantation sacs. Gross dissection revealed multiple implantation sites clustered in a single uterine horn (Fig. 2F), consistent with the expected deposition pattern of TCET. Quantitative analysis of implantation and live birth rates showed no significant differences between groups (Fig. 2E). Specifically, the live birth rate was 71.6% in the conventional group and 69.6% in the DW group. These findings indicate that DW supports normal implantation and full-term development.

### Neonatal development and organ histology

**Figure 3.**
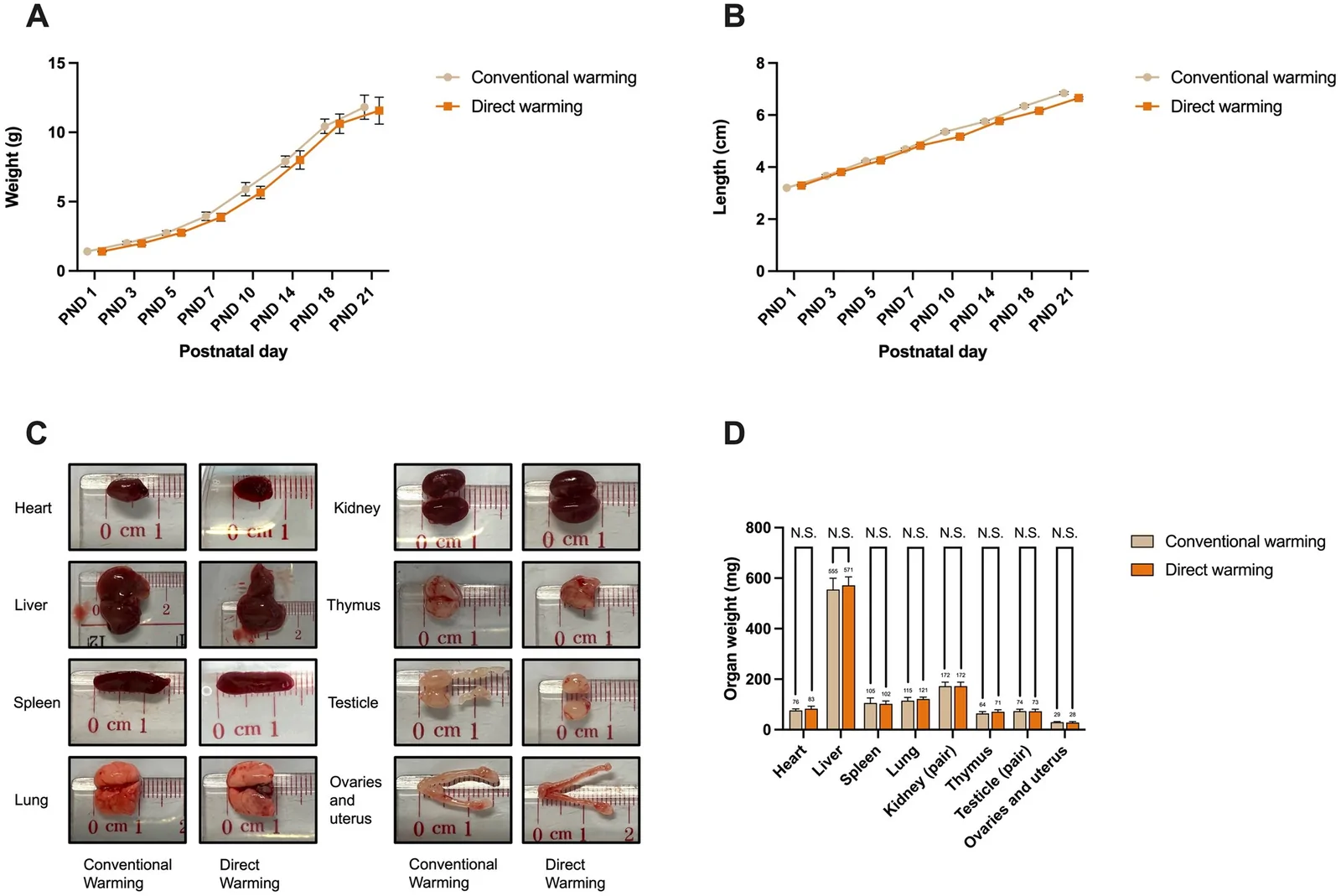
**Postnatal growth and organ development in mouse offspring derived from embryos warmed by conventional or direct methods.** (**A**) Body weight and (**B**) crown-rump length measurements of pups from postnatal day (PND) 1 to PND 21. (**C**) Representative images of major organs collected at PND 23, including heart, liver, spleen, lung, kidney, thymus, testicle, and ovaries with uterus. (**D**) Organ weights at PND 23 in both groups. Each organ was weighed individually, and paired organs were averaged. Data in (A and B) are presented as mean±SEM. Data in (D) are shown as mean values; ns = not significant. Statistical comparisons were performed using Student’s *t*-test. No significant differences were observed between the two groups across all growth and organ metrics.

To evaluate postnatal status, offspring were followed to PND 21 (Flurkey, 2009; Turgeon and Meloche, 2009; Parkinson *et al.*, 2011; Capas-Peneda *et al.*, 2021). Birth weight and subsequent growth trajectory at PND1, PND10, and PND21 were recorded (Fig. 3A and B). All physiological and behavioral milestones—including skin pigmentation, eye opening, and weaning—were achieved within expected time windows without delays (Supplementary Table S3).

At PND 23, the weights of major organs showed no significant differences between conventional and DW groups, and macroscopic examination revealed normal morphology (Fig. 3C and D). Histological analysis of 71 pups (32 conventional, 39 DW) confirmed preserved tissue architecture in all examined organs (heart, liver, spleen, lung, kidney, thymus, testis, and ovary). No pathological abnormalities were observed (Supplementary Figs S1, S2, S3, S4, S5, S6, S7, and S8), suggesting that DW does not compromise postnatal organ development.

### Comparative transcriptomic analysis reveals no substantial warming method-specific expression perturbations in cleavage-stage embryos in both human and mouse

**Figure 4.**
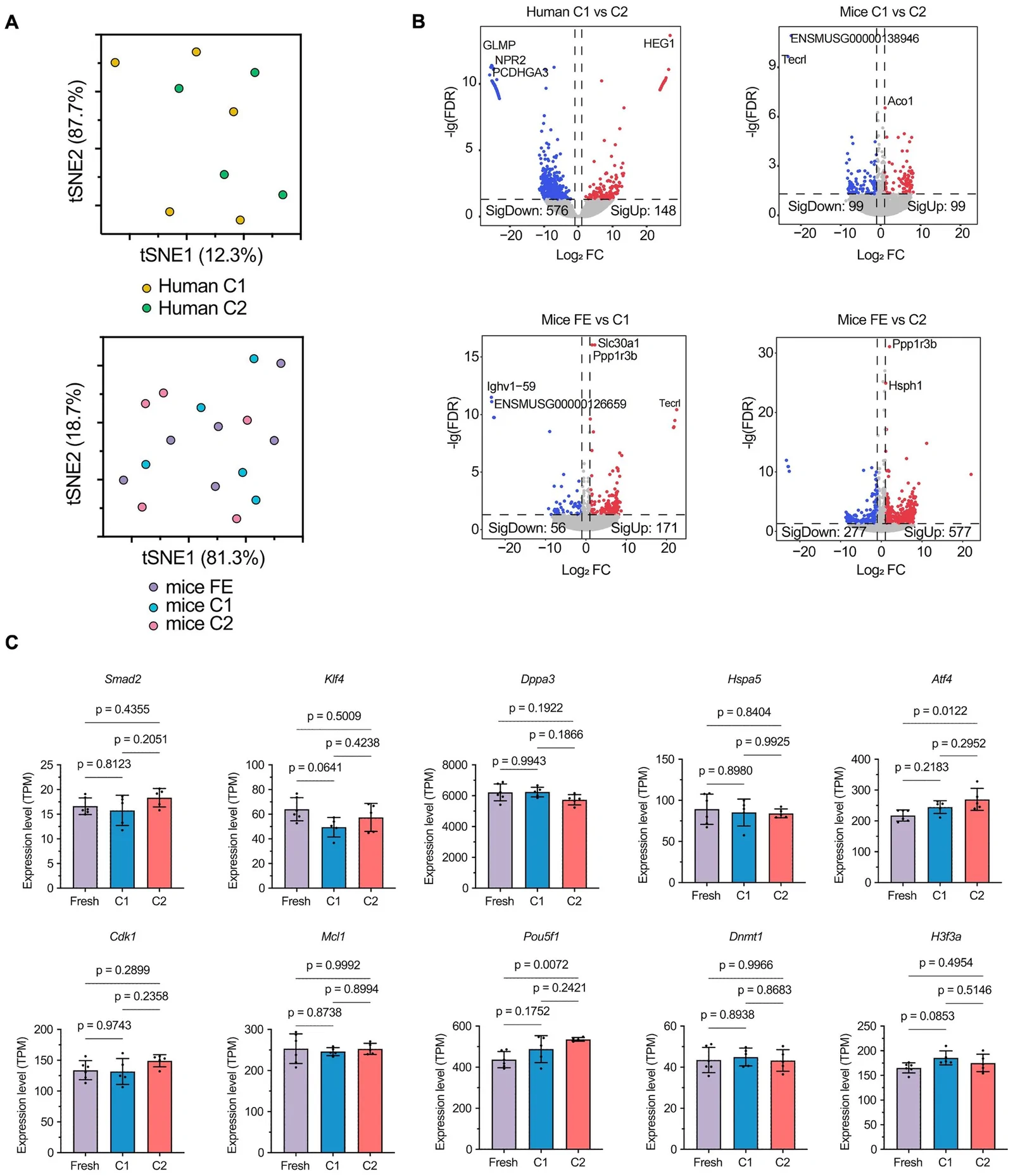
**RNA-seq profile of mice and human cleavage-stage embryos under different warming methods.** (**A**) t-SNE plot of sample clustering based on gene expression profiles. Each point represents one embryo: purple dots (mice FE), blue dots (mice C1), and red dots (mice C2); yellow dots (human C1) and green dots (human C2). (**B**) Differential gene expression analysis using volcano plots. Significantly up-regulated (red) and down-regulated (blue) genes are shown for each comparison (FDR < 0.05, |log_2_FC| > 1). Human C1 versus C2 extended analysis: 724 DEGs (148 up, 576 down). Mice C1 versus C2: 198 DEGs (99 up, 99 down). Mice FE versus C1: 227 DEGs (171 up, 56 down). Mice FE versus C2: 854 DEGs (577 up, 277 down). Selected genes with notable expression changes are labeled. (**C**) Expression levels (TPM, transcripts per million) of representative genes were compared among fresh (FE), conventional warming (C1), and direct warming (C2) groups. Bars indicate mean ± SD. *P*-values were calculated using one-way ANOVA followed by Tukey’s *post hoc* test. No statistically significant differences were observed across groups for selected genes, except for Pou5f1 (*P*=0.0072, FE vs C2). Genes were selected based on relevance to: Stress response and pluripotency pathways (*Smad2*, *Klf4*, *Dppa3*, *Hspa5*, *Atf4*), Cell cycle and apoptosis regulation (*Cdk1*, *Mcl1*, *Pou5f1*), and Epigenetic modification (*Dnmt1*, *H3f3a*).

To evaluate the molecular impact of different warming strategies, we performed integrated transcriptomic and epigenomic profiling of mouse cleavage-stage embryos (fresh FE, conventional C1, direct C2; n = 5; pools/group) and human (C1 n = 5, C2 n = 4; single embryo) cleavage-stage embryos. The corresponding morphological assessment of the human cleavage-stage embryos selected for parallel sequencing is provided in Supplementary Table S4. RNA-seq yielded high-quality data (>90% mapping, >25 000 genes in mice, >15 000 in humans) (Supplementary Fig. S9A and B). The t-SNE analysis showed intermingled clustering, indicating no systematic transcriptional shift between warming methods (Fig. 4A).

Differential expression identified limited DEGs between C1 and C2 (|log_2_FC| > 1, *P*adj < 0.05; Fig. 4B). In mice, 198 DEGs were detected, with approximately equal numbers of up- and downregulated transcripts, and in humans, 818 DEGs(576 downregulated, 148 upregulated) were identified (Supplementary Tables S5 and S6). Specific genes involved in metabolism (*Aco1*, *Tecrl*), stress (*Timp1*, *Itprip*), and development (*Smad1*) showed changes, but no GO or KEGG pathways were significantly enriched (adjusted *P* < 0.05) (Supplementary Table S7).

Compared to fresh controls, C2 showed more deviation (854 DEGs) than C1 (227 DEGs) (Fig. 4B). However, pathway analysis (e.g. ‘negative regulation of BMP signaling’, Supplementary Fig. S10A) revealed that most contributing genes exhibited only modest fold-changes and were similarly present in the FE versus C1 comparison (Supplementary Fig. S10B). This pattern indicates that the observed transcriptional alterations largely reflect generic effects of freezing-thawing rather than a distinctive consequence of the DW protocol.

To circumvent the potential biases inherent in arbitrary DEG thresholds and to capture more subtle, coordinated changes, we additionally performed GSEA across the full ranked gene lists for all pairwise comparisons (C1 vs C2, FE vs C1, FE vs C2). Consistently, this approach identified no robustly enriched pathways, confirming the absence of pathway-level transcriptional divergence between the warming methods.

Beyond global analyses, we examined a curated panel of genes with established roles in early embryogenesis in both mouse and human embryos, including pluripotency regulators (*Pou5f1*, *Klf4*, *Dppa3*), cell-cycle control (*Cdk1*), apoptosis (*Mcl1*), stress response (*Hspa5*, *Atf4*), and epigenetic regulation (*Dnmt1*, *H3f3a*) (Fig. 4C and Supplementary Fig. S10C). Across fresh, C1, and C2 groups, the expression of nearly all of these key regulators remained remarkably stable. The only notable exception was *Pou5f1* in mice, which was significantly upregulated in C2 embryos compared with fresh embryos (*P* = 0.0072; Fig. 4C). However, this change was not accompanied by consistent alterations in other core pluripotency or developmental genes, nor did it converge into any enriched pathway, suggesting that core transcriptional circuitry is robustly preserved under both warming protocols (Supplementary Table S7).

In summary, global transcriptomic profiles, including core developmental and stress regulators, remained highly consistent across all groups. The minimal gene-level differences between conventional and DW lacked pathway enrichment, indicating that DW does not induce biologically meaningful transcriptional perturbations.

### The embryonic epigenome is largely unaffected between different warming protocols

**Figure 5.**
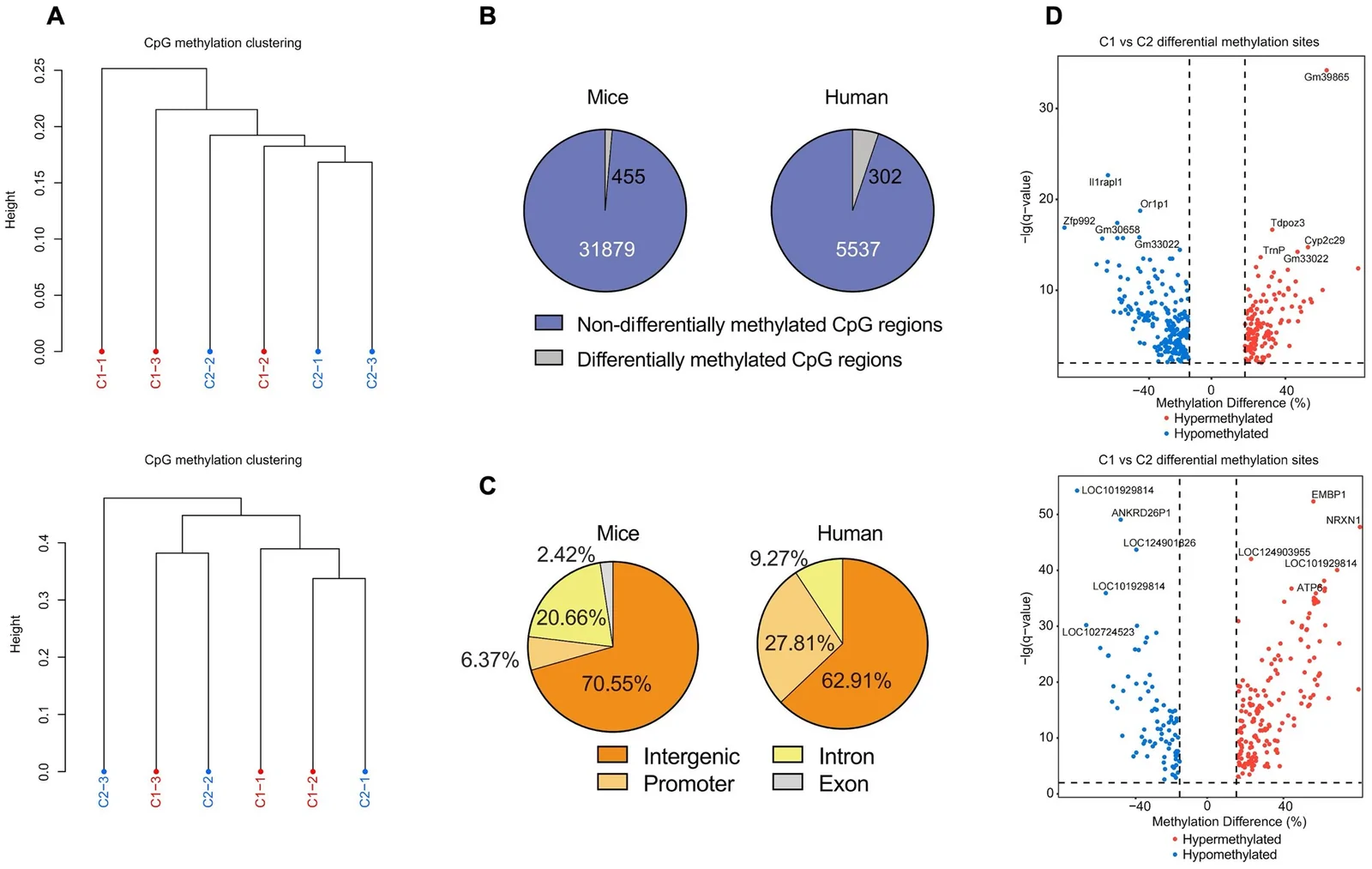
**Epigenome landscape of mice and human cleavage-stage embryos after conventional or direct warming.** (**A**) Hierarchical clustering dendrograms of CpG methylation profiles across the samples. The vertical axis (Height) represents the dissimilarity distance between samples in arbitrary units, reflecting the degree of variance in global methylation levels between groups (C1 and C2). (**B**) Pie chart showing the proportion of mice (left) and human (right) embryos DMR between C1 and C2 conditions. (**C**) Pie chart showing the distribution of DMRs across genomic features. Percentages indicate the proportion of DMRs in each category. (**D**) Volcano plot showing annotated DMRs between C1 and C2 methods in mice and human. Red and blue dots represent hypermethylated and hypomethylated regions in C2 relative to C1, respectively. Selected regions with substantial methylation differences are annotated. DMRs were identified based on thresholds of absolute methylation difference > 25% and adjusted *P*-value (*q*-value) < 0.01.

To investigate whether different warming methods affect the embryonic methylome, we performed WGBS-seq on separated nuclei from mouse and human embryos. Both species exhibited highly stable M-bias profiles and comparable global methylation levels across warming method (Supplementary Fig. S9C). Consistently, biological replicates exhibited high intra-group correlations (r = 0.83–0.89), with C2 embryos showing slightly greater internal consistency (Supplementary Fig. S9D). Furthermore, CpG methylation-based clustering revealed no method-specific grouping in either species (Fig. 5A). Locus-specific analysis identified few DMRs (mouse: 456; human: 303), with mouse DMRs confined to non-functional elements and human DMRs rarely locating to promoter (7.66%) (Fig. 5B and C). Annotation of these regions identified a limited number of DMGs: 195 in mice (from 341 DMRs; 133 hyper-, 208 hypomethylated) and 50 in humans (from 262 DMRs; 178 hyper-, 83 hypomethylated) (Fig. 5D). However, none of these DMGs showed significant enrichment across GO, KEGG, GSEA, Reactome, or ChEA3 transcription factors analyses (Supplementary Table S8).

Collectively, these results demonstrate that DW supports normal post-warming development, implantation, and neonatal outcomes in mice and yields largely stable transcriptomic and epigenomic profiles in both murine and human cleavage-stage embryos, despite minor molecular differences that lack pathway-level significance.

## Discussion

This study systematically evaluated the safety and efficacy of DW, a highly simplified embryo warming method using a standard culture medium, in comparison to the conventional stepwise protocol. Preclinical validation using a murine model confirmed that DW fully maintained post-warming embryonic competence and supported normal *in vivo* development through to weaning. Crucially, the streamlined protocol resulted in embryo survival, implantation, and live birth rates entirely comparable to those of conventional warming. Moreover, offspring from the DW group exhibited normal growth patterns, timely developmental milestones, and unremarkable histological morphology of key organs, suggesting that the streamlined method does not compromise early postnatal development.

Although eliminating sequential dilution steps accelerates embryo rehydration and potentially increases osmotic stress, our findings demonstrate that cleavage-stage embryos tolerate this abrupt transition. This robust tolerance is likely facilitated by the rapid efflux of highly permeating CPAs used in both murine (DMSO and propylene glycol) (Nakao *et al.*, 1997) and human (dimethyl sulfoxide and ethylene glycol) (Konc *et al.*, 2014) vitrification protocols, which minimizes intracellular osmotic shifts and swelling-induced lysis (Bhattacharya, 2018).

By controlling all experimental conditions aside from the DW method, this study enabled a robust comparison of post-warm viability. Live birth was considered a clinically relevant endpoint (Barnhart, 2014), and no adverse effects were observed in either implantation success or early postnatal development. However, the mouse model’s physiological differences, such as smaller embryo size and distinct membrane properties, may limit generalizability to humans (Lee *et al.*, 2015). Larger-scale randomized controlled trials in humans are now supported by the government research funds and conducted in our ART center.

Importantly, this study extends previous work that focused primarily on *in vitro* endpoints such as survival, re-expansion, and blastocyst development (Karagianni *et al.*; Jiang *et al.*, 2024; Leisinger *et al.*, 2024; Liebermann *et al.*, 2024; Ezoe *et al.*, 2024; Fucci *et al.*, 2025; Lammers *et al.*, 2025; Licata *et al.*, 2025; Shioya *et al.*, 2025), by offering *in vivo* data on neonatal development. Although some mouse studies have explored implantation and fetal morphology after embryo warming, postnatal organ-level assessments remain limited (Larman and Gardner, 2011; Jin and Mazur, 2015; Taketsuru *et al.*, 2022; Chen *et al.*, 2024). Our results fill this gap by demonstrating comparable gross and histological features in major organs of offspring from both warming groups. These findings, together with the known biophysical advantages of high-permeability CPAs in reducing ice recrystallization (Takahashi *et al.*, 1985; Edashige, 2016; García-Martínez *et al.*, 2021; Ahmadkhani *et al.*, 2025), suggest that DW does not impair key developmental processes.

Because physiological normality can mask subtle molecular shifts, we further validated the molecular safety of DW through comprehensive transcriptomic and methylomic profiling.

Focusing first on the transcriptome, our data from both murine and human cleavage-stage embryos confirm that DW broadly preserves global gene expression integrity. Consistent with expectations, the act of cryopreservation itself introduces baseline transcriptional variance, as evidenced by the larger sets of DEGs when comparing fresh and warmed embryos. Crucially, the choice between conventional and DW protocols exerted minimal biological impact. Although direct comparison between the two warming methods identified a small subset of DEGs related to metabolism and structural organization, these genes did not yield significantly enriched functional pathways. Furthermore, targeted evaluation of core developmental, apoptotic, and stress-response regulators revealed remarkably stable expression across both species. Together, these data suggest that the rapid physical transition of DW does not induce overt or coherent disruption of the essential transcriptional programs required for early development.

Parallel to the transcriptomic findings, our methylome data illustrate a largely unperturbed epigenetic landscape under both warming protocols. In both murine and human models, clustering analyses demonstrated high intra-group similarity without segregation based on the warming method. While DMRs were detected—with humans exhibiting expectedly higher biological variability and a greater number of DMRs than mice—the vast majority mapped to intergenic or intronic regions. Crucially, subsequent annotation of DMGs failed to identify significant enrichment in major biological pathways or transcription factor networks. This indicates that, at the level of gene-associated DNA methylation, the streamlined warming protocol does not trigger systemic epigenetic reprogramming.

Placing our human methylomic data into the broader developmental context further reinforces these safety conclusions. Specifically, global 5mC typically declines from ∼41% (zygotes) to ∼29% (blastocysts) (Guo *et al.*, 2014), while cleavage-to-morula stages averaging ∼35.1% despite transient 8-cell *de novo* methylation (Zhu *et al.*, 2018). Within our cohort, directly warmed embryos displayed a wider spread of methylation levels, including one sample exceeding 50%. Rather than indicating a protocol-induced abnormality, this outlier likely reflects the inherent biological heterogeneity and variable stress-response capacities characteristic of early human embryogenesis. Given the overall lack of pathway enrichment among DMGs, we interpret this variation as intrinsic rather than a specific detrimental consequence of the DW procedure.

Despite the reassuring findings, several limitations should be considered. First, although mouse model enables controlled experimentation and vital *in vivo* postnatal assessments, inherent species-specific differences, such as distinct membrane properties and developmental timelines, restrict direct extrapolation to clinical human ART. Second, the animal trials lacked formal randomization and blinding, although our reliance on highly objective endpoints, including survival rates and organ histology, helps mitigate potential observer bias. Finally, while transcriptomic and DNA methylation provide robust insights, they do not capture the entire molecular phenotype. Future studies incorporating complementary omics layers, such as proteomics, metabolomics, chromatin accessibility, or RNA modifications, could offer more holistic understanding on how warming strategies impact human embryo physiology. Ultimately, the large-scale randomized controlled trials currently underway at our center will be essential to definitively establish the clinical non-inferiority and broader applicability of this streamlined protocol.

## Conclusion

In summary, this study provides preclinical and molecular evidence supporting the safety and feasibility of DW. By demonstrating comparable developmental outcomes and stable transcriptomic and methylomic landscapes in both mouse and human embryos, DW emerges as a promising and simplified alternative to conventional warming protocols in both mouse model and human.

## Supplementary Material

deag105_Supplementary_Materials_and_Methods

deag105_Supplementary_Figure_S1

deag105_Supplementary_Figure_S2

deag105_Supplementary_Figure_S3

deag105_Supplementary_Figure_S4

deag105_Supplementary_Figure_S5

deag105_Supplementary_Figure_S6

deag105_Supplementary_Figure_S7

deag105_Supplementary_Figure_S8

deag105_Supplementary_Figure_S9

deag105_Supplementary_Figure_S10

deag105_Supplementary_Table_S1

deag105_Supplementary_Table_S2

deag105_Supplementary_Table_S3

deag105_Supplementary_Table_S4

deag105_Supplementary_Table_S5

deag105_Supplementary_Table_S6

deag105_Supplementary_Table_S7

deag105_Supplementary_Table_S8

## Data Availability

The datasets generated during and/or analyzed during the current study are available from the corresponding author upon reasonable request.
