# Supplementary material for: Multi-omics and developmental comparison of direct and conventional warming methods in vitrified human and mouse cleavage-stage embryos
Source: Hum Reprod. 2026 Jul 6;41(9):1522–33. doi: 10.1093/humrep/deag105 (PMC13550675; doi:10.1093/humrep/deag105)
Supplement: Supplementary file 1 [file deag105_supplementary_figure_s1.pdf]

Human Reproduction, 2026, 1–1
https://doi.org/10.1093/humrep/deag105
Supplementary Data

human reproduction

OXFORD

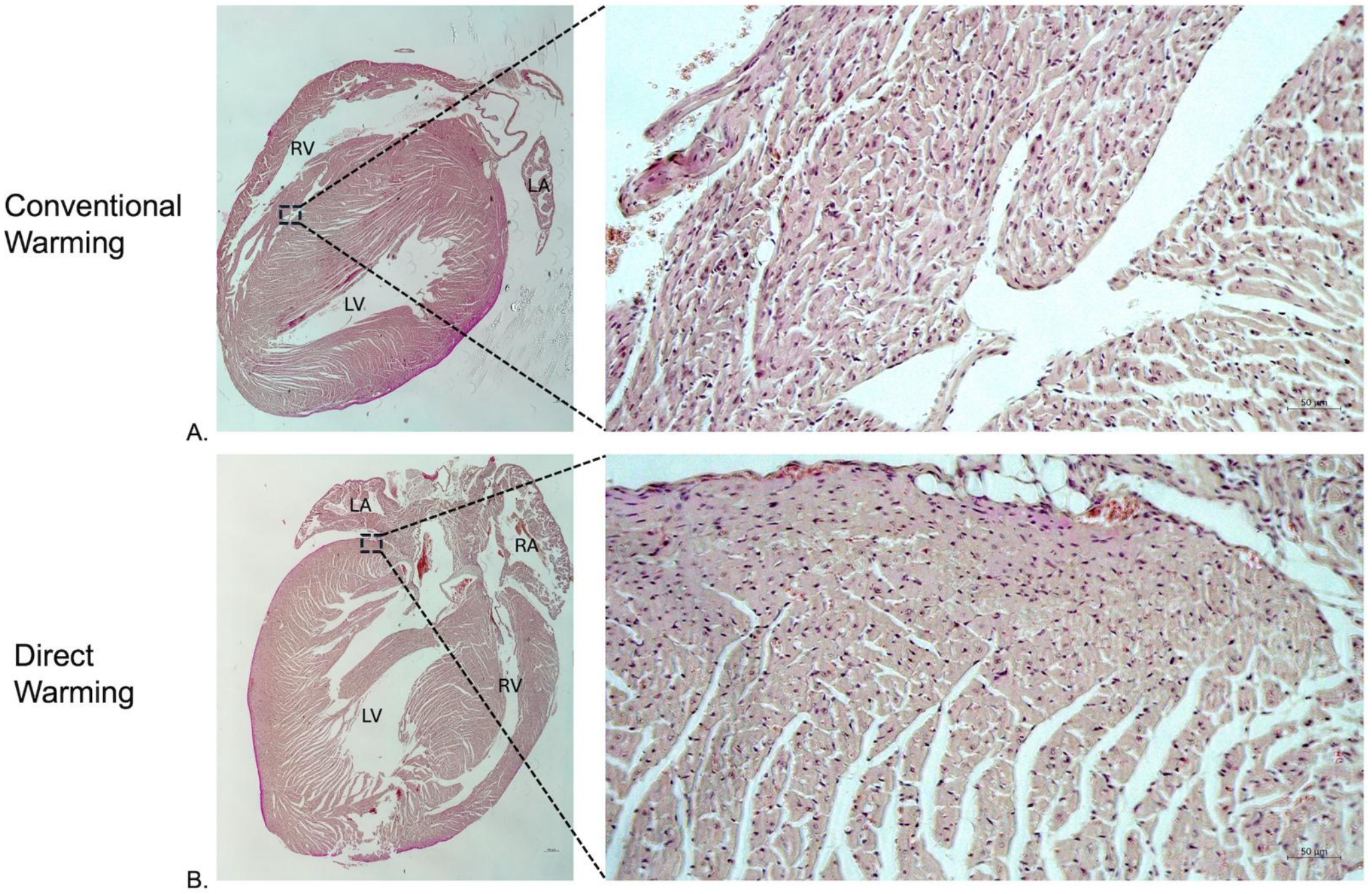

Supplementary Figure S1. Representative H&E-stained heart sections from neonatal mice in conventional and direct warming groups: stitched overview and 20× magnification. (**A**) Conventional warming group and (**B**) direct warming group. Left panels show stitched whole-section overviews (LA: left atrium, RA: right atrium, LV: left ventricle, RV: right ventricle); right panels display selected regions at 20× magnification. Normal cardiac histology is characterized by tightly packed, striated cardiomyocytes with centrally located nuclei and organized alignment. No structural abnormalities or group differences were observed, indicating preserved cardiac morphology across both embryo warming methods.
