# Supplementary material for: Multi-omics and developmental comparison of direct and conventional warming methods in vitrified human and mouse cleavage-stage embryos
Source: Hum Reprod. 2026 Jul 6;41(9):1522–33. doi: 10.1093/humrep/deag105 (PMC13550675; doi:10.1093/humrep/deag105)
Supplement: Supplementary file 2 [file deag105_supplementary_figure_s10.pdf]

Human Reproduction, 2026, 1–1
https://doi.org/10.1093/humrep/deag105
Supplementary Data

human reproduction
OXFORD

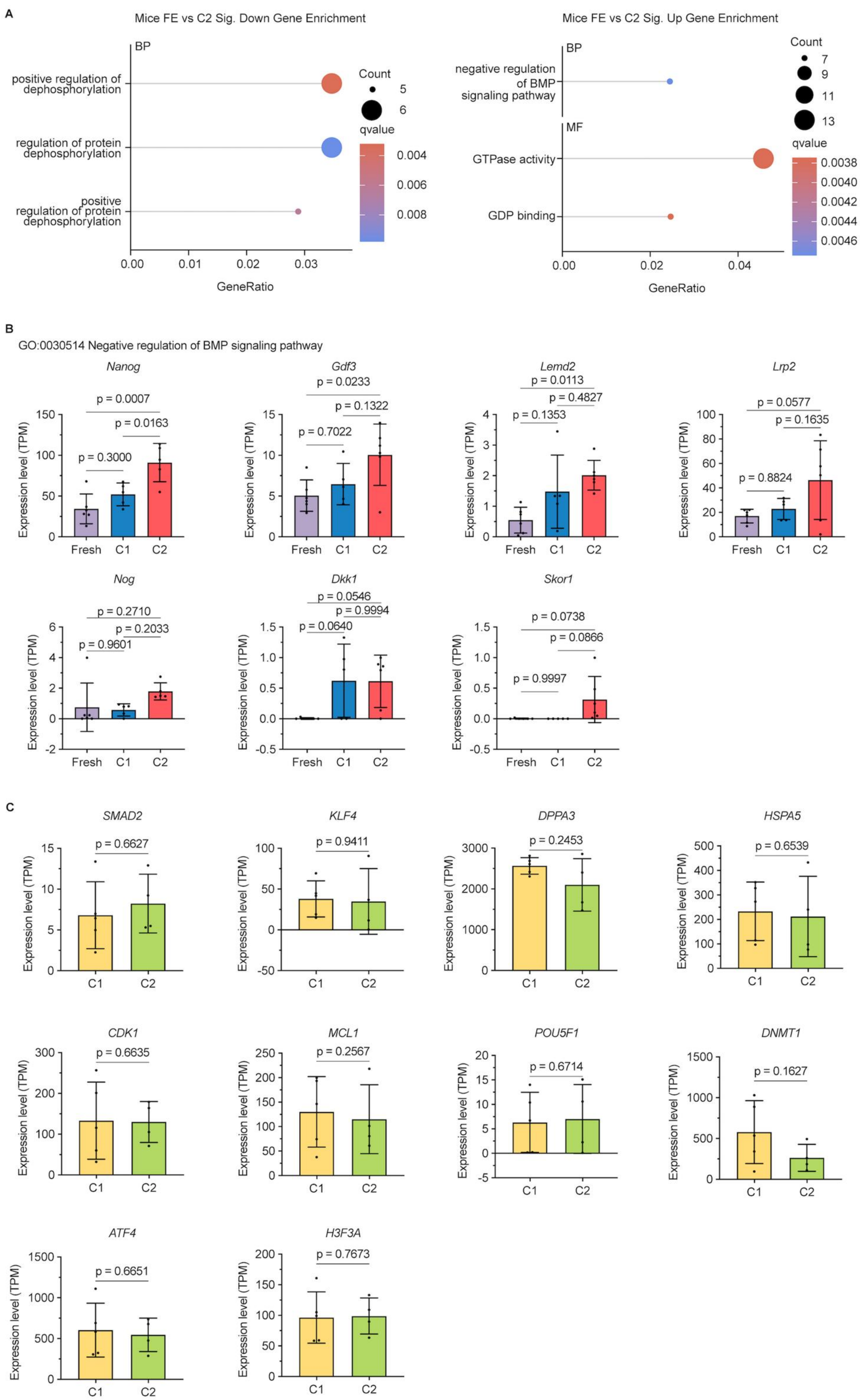

Supplementary Figure S10. Functional enrichment analysis and developmental critical genes expression in mouse and human embryos under different warming methods. (**A**) GO enrichment analysis of down-regulated (blue) genes and up-regulated (red) in mice FE versus C2. Bubble size represents gene count and color intensity indicates adjusted *P*-value significance. Only significantly enriched terms (q-value < 0.01) are shown. (**B**) Expression levels (TPM) of differentially expressed genes associated with negative regulation of BMP signaling pathway. Data presented as mean ± SD. (**C**) Expression levels of key genes in human embryos (reproduced from Fig. 5 for comparison).
