# Supplementary material for: Multi-omics and developmental comparison of direct and conventional warming methods in vitrified human and mouse cleavage-stage embryos
Source: Hum Reprod. 2026 Jul 6;41(9):1522–33. doi: 10.1093/humrep/deag105 (PMC13550675; doi:10.1093/humrep/deag105)
Supplement: Supplementary file 3 [file deag105_supplementary_figure_s2.pdf]

Human Reproduction, 2026, 1–1
https://doi.org/10.1093/humrep/deag105
Supplementary Data

human reproduction

OXFORD

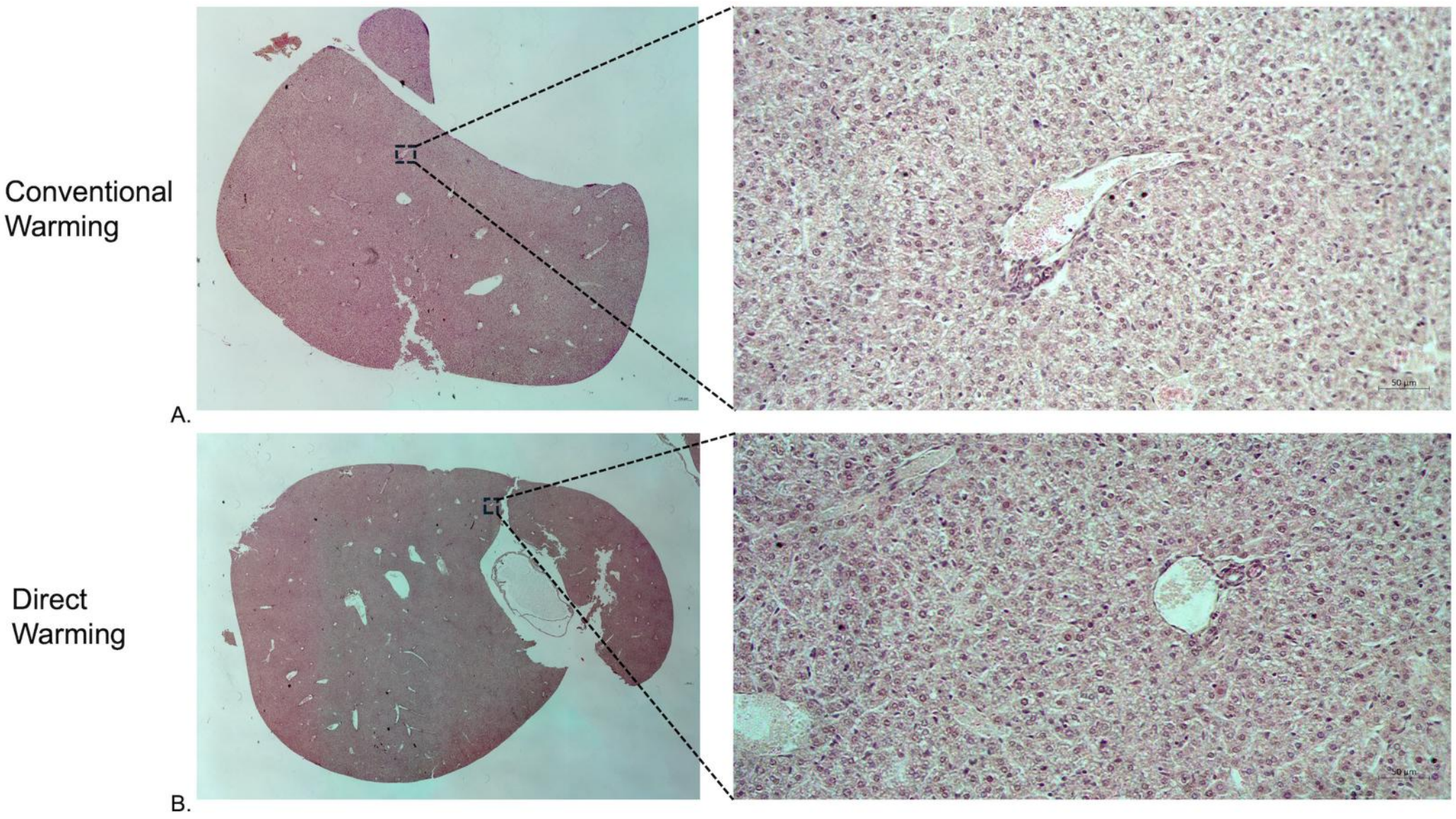

Supplementary Figure S2. Representative H&E-stained liver sections from neonatal mice in conventional and direct warming groups: stitched overview and 20× magnification. (**A**) Conventional warming group and (**B**) direct warming group. Left panels show stitched overviews of entire liver lobes from postnatal day 23 (PND 23) mice; right panels display selected regions at 20× magnification. Normal hepatic architecture includes radially arranged hepatic cords, centrally located veins, and evenly distributed hepatocytes with round nuclei and eosinophilic cytoplasm. No histological abnormalities or intergroup differences were observed.
