# Supplementary material for: Multi-omics and developmental comparison of direct and conventional warming methods in vitrified human and mouse cleavage-stage embryos
Source: Hum Reprod. 2026 Jul 6;41(9):1522–33. doi: 10.1093/humrep/deag105 (PMC13550675; doi:10.1093/humrep/deag105)
Supplement: Supplementary file 4 [file deag105_supplementary_figure_s3.pdf]

Human Reproduction, 2026, 1–1
https://doi.org/10.1093/humrep/deag105
Supplementary Data

human reproduction

OXFORD

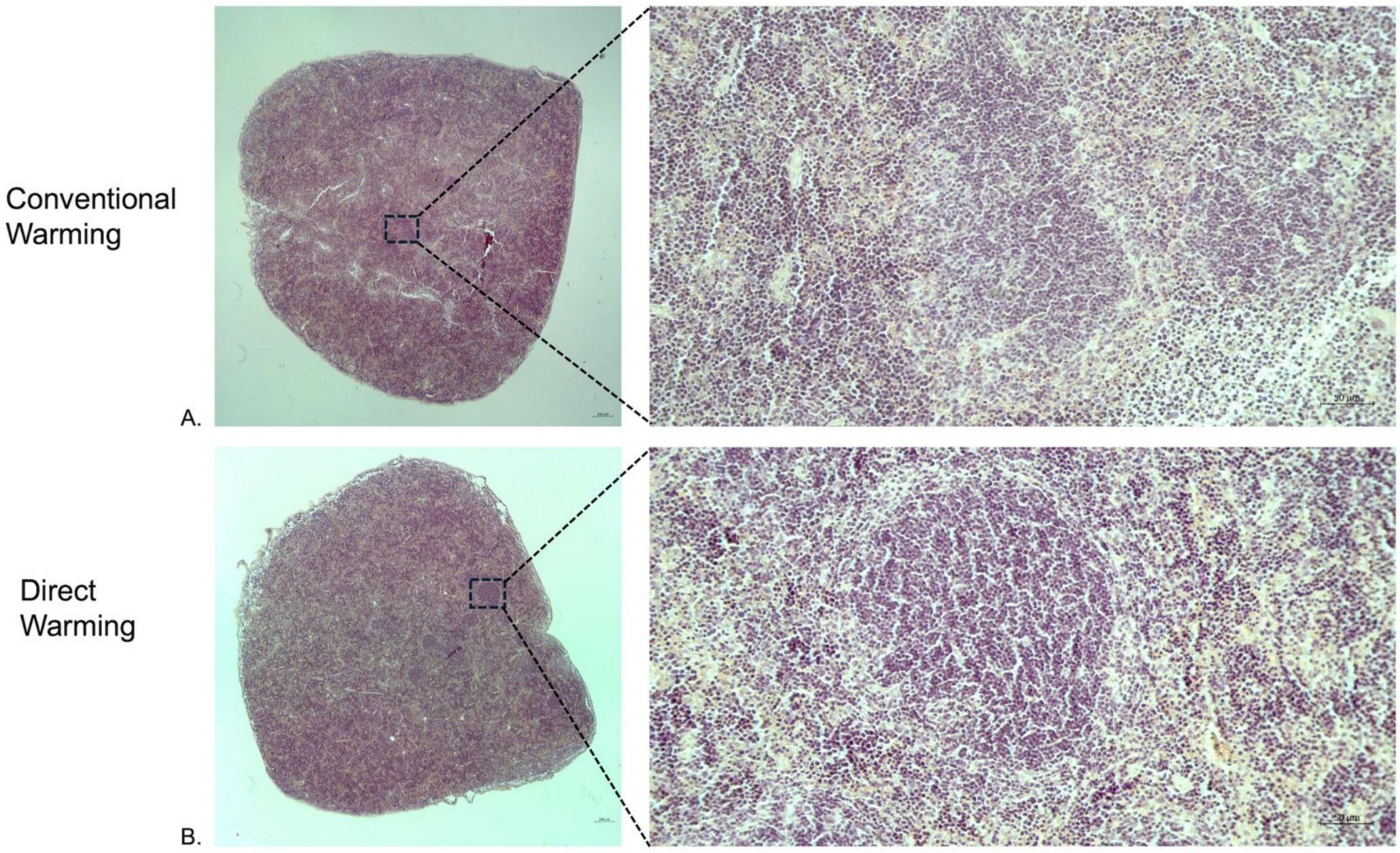

Supplementary Figure S3. Representative H&E-stained spleen sections from neonatal mice in conventional and direct warming groups: stitched overview and 20× magnification. (**A**) Conventional warming group and (**B**) direct warming group. H&E-stained spleen sections from PND 23 mice are shown, with stitched low-magnification overviews on the left and 20× magnified regions on the right. Normal splenic architecture includes well-demarcated white pulp (lymphoid follicles) surrounded by red pulp, with distinct marginal zones. No morphological differences were observed between the two groups, indicating normal splenic development.
