# Supplementary material for: Multi-omics and developmental comparison of direct and conventional warming methods in vitrified human and mouse cleavage-stage embryos
Source: Hum Reprod. 2026 Jul 6;41(9):1522–33. doi: 10.1093/humrep/deag105 (PMC13550675; doi:10.1093/humrep/deag105)
Supplement: Supplementary file 6 [file deag105_supplementary_figure_s5.pdf]

Human Reproduction, 2026, 1–1
https://doi.org/10.1093/humrep/deag105
Supplementary Data

human reproduction
OXFORD

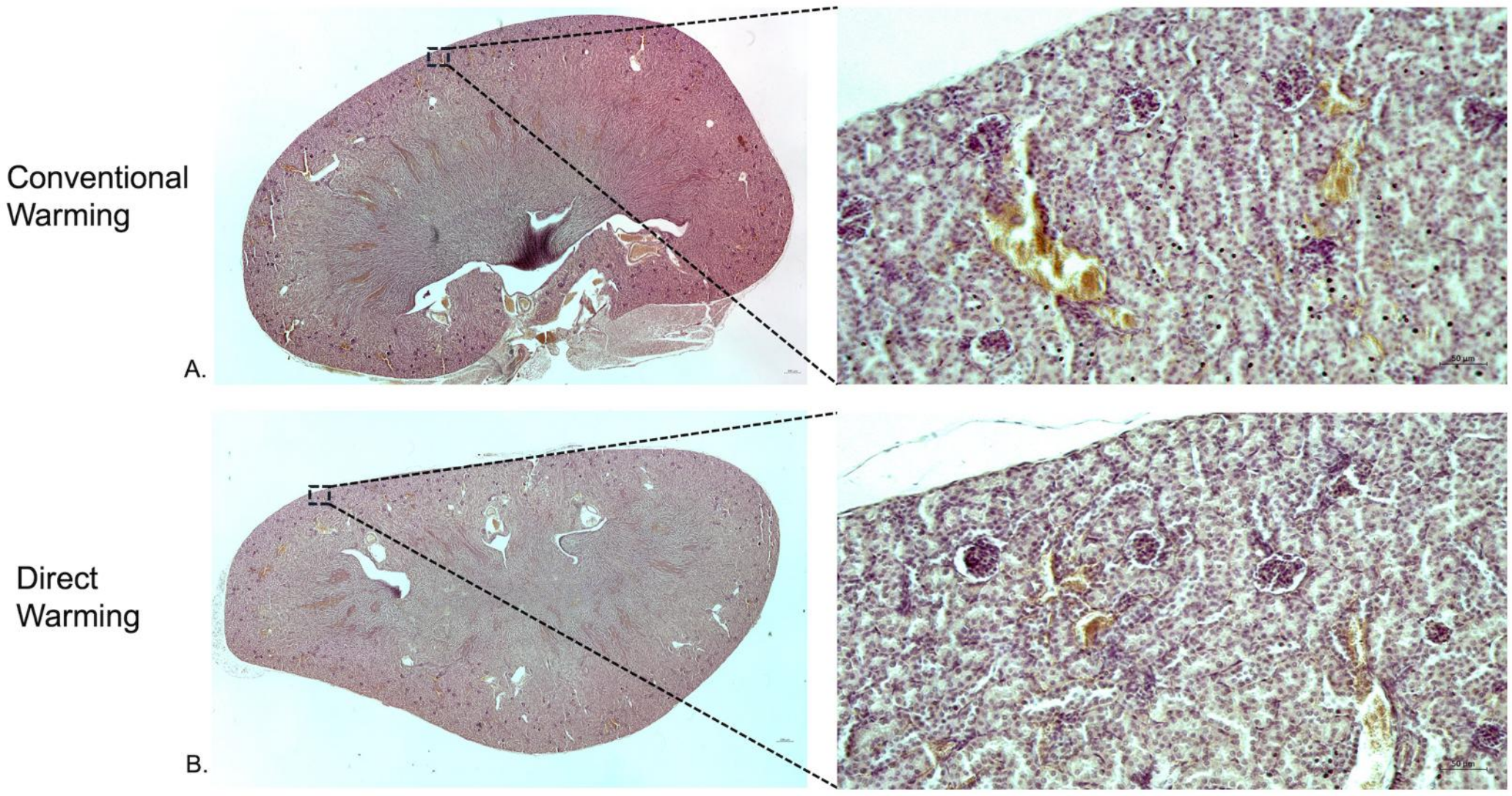

Supplementary Figure S5. Representative H&E-stained kidney sections from neonatal mice in conventional and direct warming groups: stitched overview and 20× magnification. (**A**) Conventional warming group and (**B**) direct warming group. Kidney sections from PND 23 mice are presented with stitched overview images on the left and 20× magnified regions on the right. Normal renal histology includes well-organized cortical and medullary regions, clearly defined renal corpuscles with glomeruli, and aligned proximal and distal tubules. No structural abnormalities or differences were observed between the two groups, indicating comparable postnatal renal development.
