# Supplementary material for: Multi-omics and developmental comparison of direct and conventional warming methods in vitrified human and mouse cleavage-stage embryos
Source: Hum Reprod. 2026 Jul 6;41(9):1522–33. doi: 10.1093/humrep/deag105 (PMC13550675; doi:10.1093/humrep/deag105)
Supplement: Supplementary file 7 [file deag105_supplementary_figure_s6.pdf]

Human Reproduction, 2026, 1–1
https://doi.org/10.1093/humrep/deag105
Supplementary Data

human reproduction
OXFORD

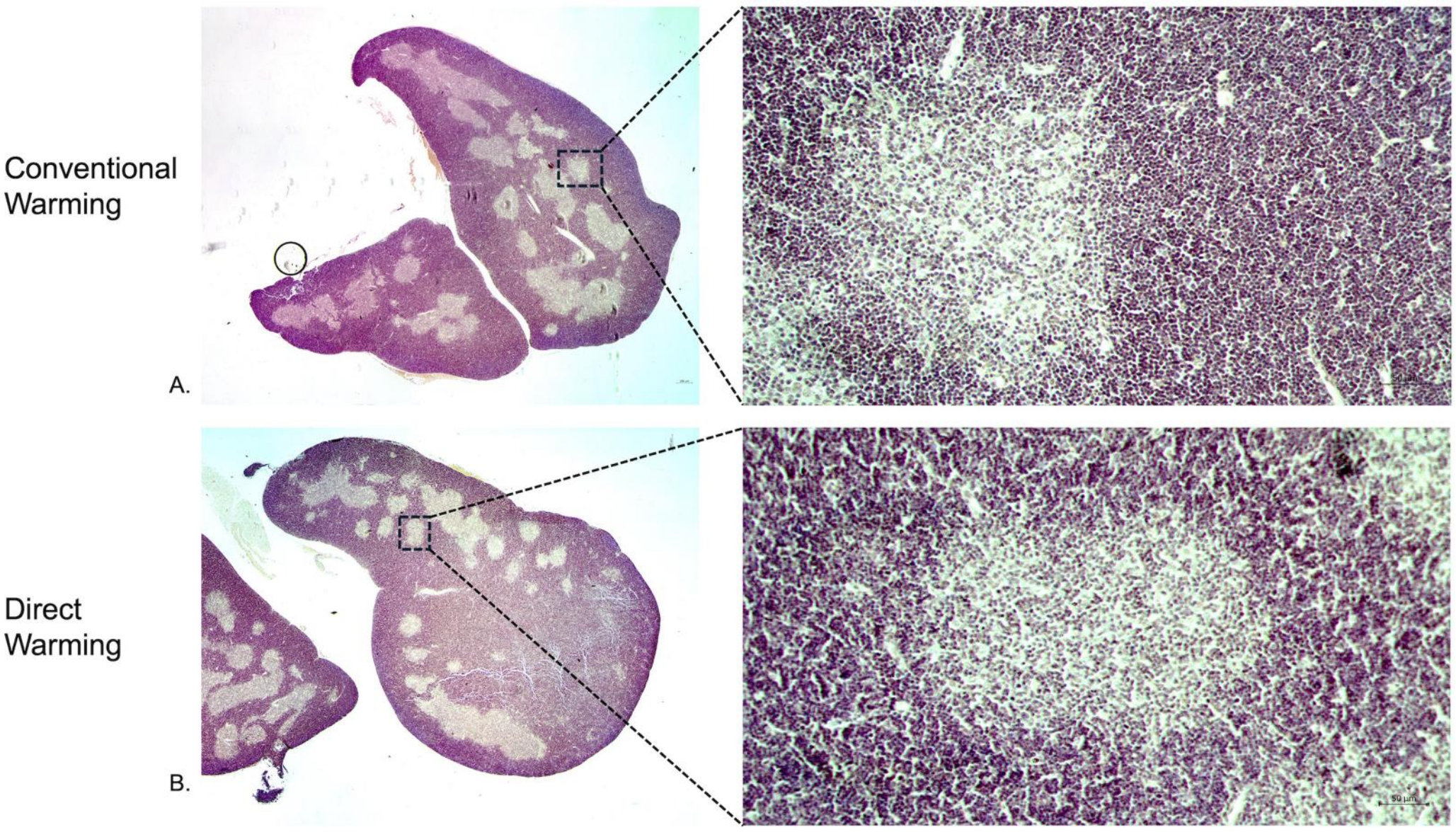

Supplementary Figure S6. Representative H&E-stained thymus sections from neonatal mice in conventional and direct warming groups: stitched overview and 20× magnification. (**A**) Conventional warming group and (**B**) direct warming group. Thymus sections from PND 23 mice are shown with stitched low-magnification overviews (left) and 20× magnified regions (right). Normal thymic architecture displays distinct cortical and medullary zones, with densely packed lymphocytes in the cortex and lighter-staining medullary regions. No histological abnormalities or intergroup differences were observed, indicating normal immune organ development following both embryo warming methods.
