# Supplementary material for: Multi-omics and developmental comparison of direct and conventional warming methods in vitrified human and mouse cleavage-stage embryos
Source: Hum Reprod. 2026 Jul 6;41(9):1522–33. doi: 10.1093/humrep/deag105 (PMC13550675; doi:10.1093/humrep/deag105)
Supplement: Supplementary file 8 [file deag105_supplementary_figure_s7.pdf]

Human Reproduction, 2026, 1–1
https://doi.org/10.1093/humrep/deag105
Supplementary Data

human reproduction
OXFORD

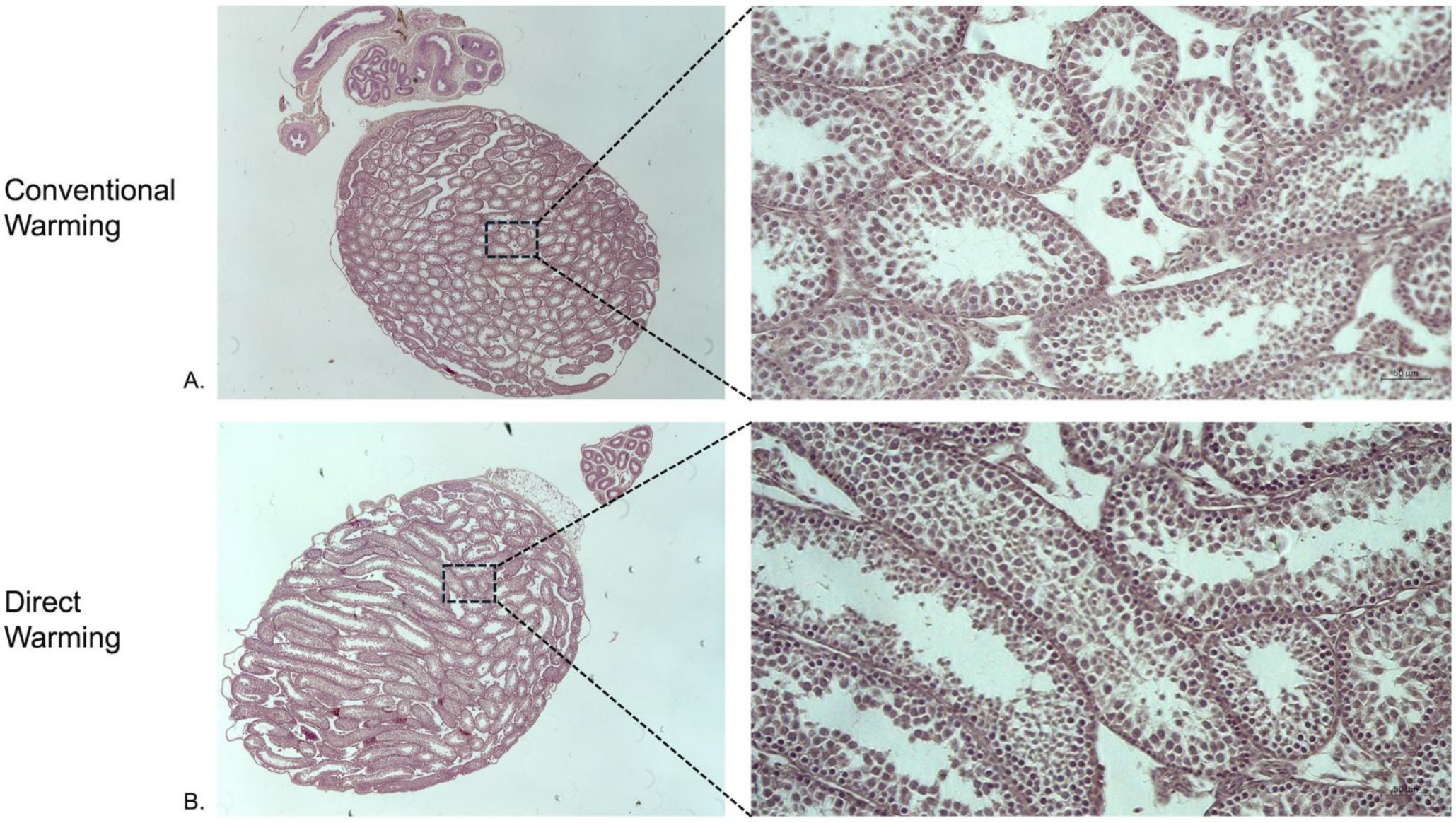

Supplementary Figure S7. Representative H&E-stained testicle sections from neonatal mice in conventional and direct warming groups: stitched overview and 20× magnification. (**A**) Conventional warming group and (**B**) direct warming group. Testicular sections from PND 23 mice are shown with stitched overview images on the left and 20× magnified regions on the right. Normal testicular histology includes developing seminiferous tubules lined with spermatogonia and Sertoli cells, surrounded by interstitial tissue containing Leydig cells. No structural differences were observed between groups, indicating normal early postnatal testicular development.
