# Supplementary material for: Multi-omics and developmental comparison of direct and conventional warming methods in vitrified human and mouse cleavage-stage embryos
Source: Hum Reprod. 2026 Jul 6;41(9):1522–33. doi: 10.1093/humrep/deag105 (PMC13550675; doi:10.1093/humrep/deag105)
Supplement: Supplementary file 9 [file deag105_supplementary_figure_s8.pdf]

Human Reproduction, 2026, 1–1
https://doi.org/10.1093/humrep/deag105
Supplementary Data

human reproduction
OXFORD

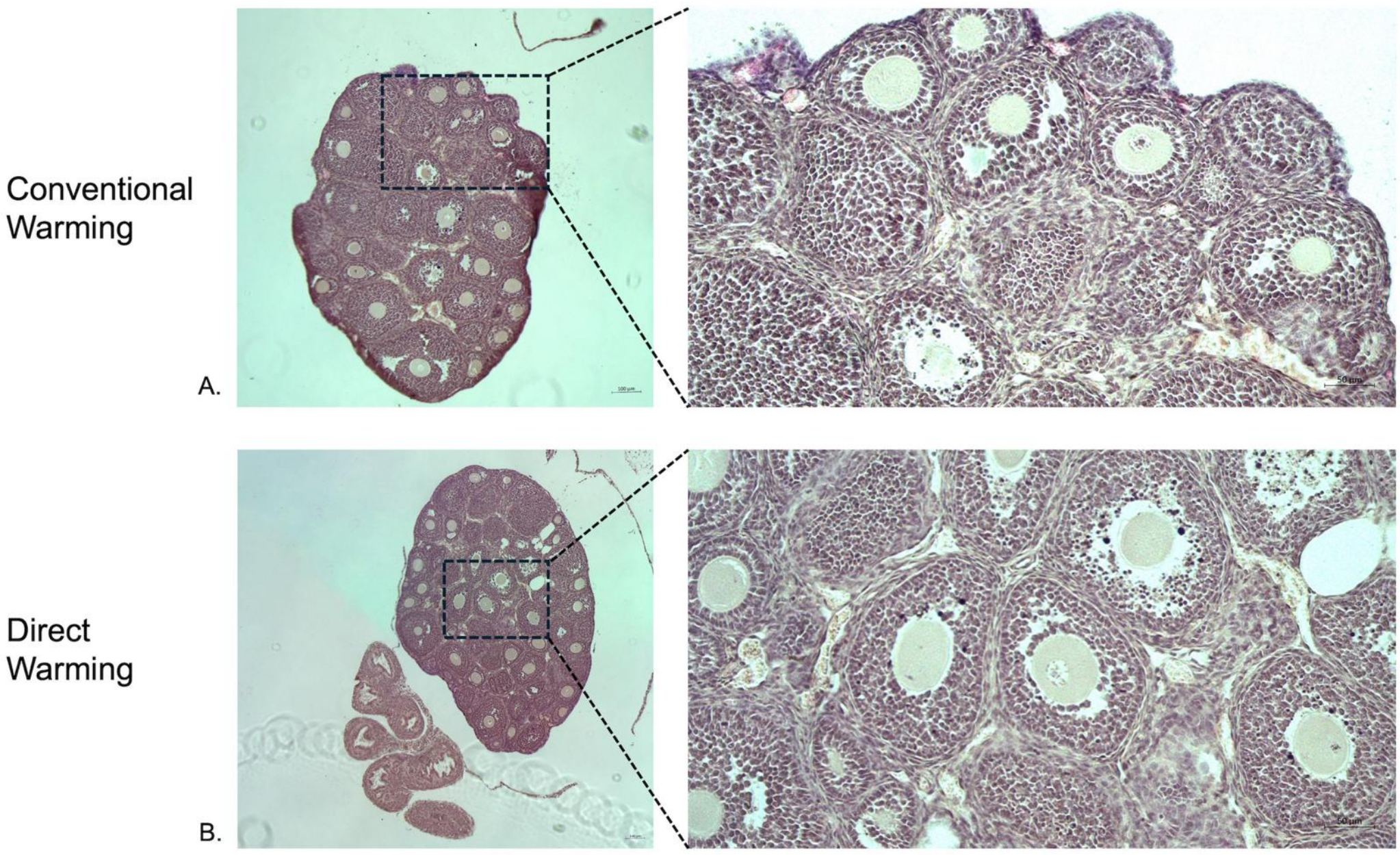

Supplementary Figure S8. Representative H&E-stained ovary sections from neonatal mice in conventional and direct warming groups: stitched overview and 20× magnification. (**A**) Conventional warming group and (**B**) direct warming group. Ovary sections from PND 23 mice are displayed with stitched low-magnification overviews on the left and 20× magnified views on the right. Normal ovarian morphology includes a cortical region containing numerous primordial and primary follicles, and a medullary region composed of stromal tissue and blood vessels. No histological abnormalities or differences were observed between groups, suggesting comparable ovarian development following both warming methods.
