# Supplementary material for: Multi-omics and developmental comparison of direct and conventional warming methods in vitrified human and mouse cleavage-stage embryos
Source: Hum Reprod. 2026 Jul 6;41(9):1522–33. doi: 10.1093/humrep/deag105 (PMC13550675; doi:10.1093/humrep/deag105)
Supplement: Supplementary file 10 [file deag105_supplementary_figure_s9.pdf]

*Human Reproduction*, 2026, 1–1
https://doi.org/10.1093/humrep/deag105
**Supplementary Data**

human reproduction
OXFORD

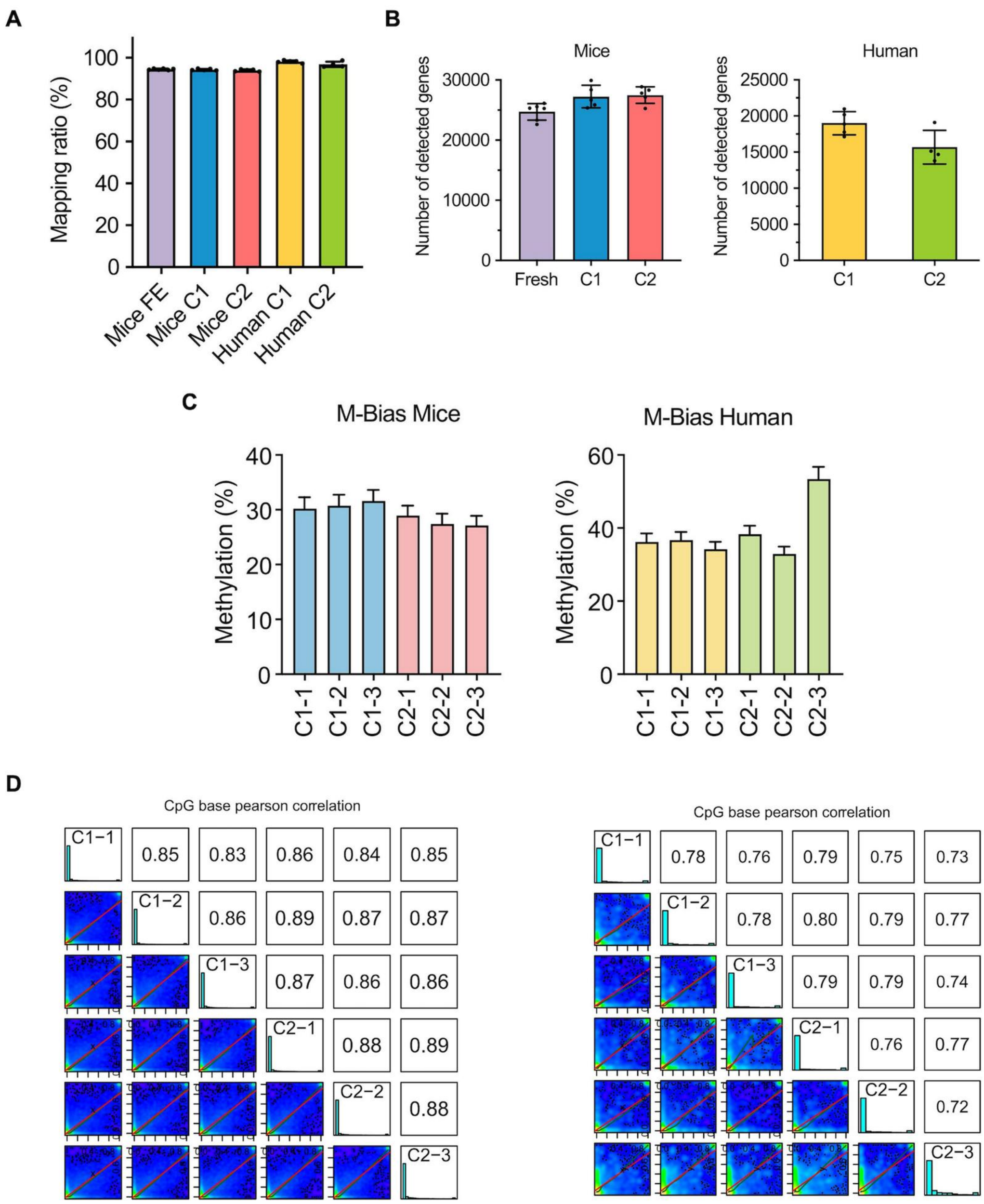


**Supplementary Figure S9. Transcriptomic quality control and global DNA methylation baselines in mouse and human embryos following different warming methods.** (**A**) RNA-seq mapping ratios mouse (FE, C1, and C2) and human (C1 and C2) embryo groups. FE: fresh embryos; C1: conventional warming; C2: direct warming. Data presented as mean ± SD. (**B**) Number of detected genes in each group. Data presented as mean ± SD. (**C**) Average DNA methylation bias levels across individual replicates in mouse and human embryos. Bars represent mean ± SD. (**D**) Pairwise Pearson correlation matrix showing methylation concordance among individual embryos from C1 and C2 methods in both species.
