# Supplementary material for: Multi-omics and developmental comparison of direct and conventional warming methods in vitrified human and mouse cleavage-stage embryos
Source: Hum Reprod. 2026 Jul 6;41(9):1522–33. doi: 10.1093/humrep/deag105 (PMC13550675; doi:10.1093/humrep/deag105)
Supplement: Supplementary file 11 [file deag105_supplementary_materials_and_methods.pdf]

Human Reproduction, 2026, 1–2
https://doi.org/10.1093/humrep/deag105
Supplementary Data

human reproduction
OXFORD

Detailed bioinformatics and statistical analysis pipelines

RNA-seq analysis

For transcriptome profiling, raw paired-end RNA-seq reads were first subjected to quality assessment using FastQC (v0.12.1) to evaluate base quality score distributions, GC content, sequence duplication levels, overrepresented sequences, and adapter contamination. Quality-control (QC) reports were inspected to confirm the absence of severe technical artifacts. Reads were then preprocessed using fastp (v0.23.4), which performed adapter trimming, removal of low-quality bases (default Phred quality cutoff of 20), correction of mismatched bases where applicable, and filtering of short reads (minimum read length set to 50 bp after trimming, unless stated otherwise). Post-trimming QC was re-evaluated with FastQC and summarized with MultiQC (v1.25.1) to confirm improvement in read quality and effective adapter removal.

High-quality reads were aligned to the appropriate reference genome (mouse: GRCm39; human: GRCh38) using the splice-aware aligner HISAT2 (v2.2.1) (Kim *et al.*, 2015). Indexes were built from the reference genome using the corresponding gene annotation file. Alignments were performed in paired-end mode with default parameters, rRNA reads were removed first, allowing for canonical splice junctions. Only primary alignments with mapping quality ≥ 20 were retained for downstream analysis. Alignment statistics, including overall mapping rate, properly paired read fraction, and insert-size distributions, were summarized using samtools (v1.21) and Qualimap (v2.3.2).

Gene-level quantification was performed using featureCounts (v2.0.7) from the Subread package, assigning reads to annotated exons and summarizing counts at the gene level. Multi-mapping and chimeric reads were excluded.

Differential expression analysis was carried out using DESeq2 (v1.42.0) (Love *et al.*, 2014) in R (v4.4.0). Raw counts were normalized using the median-of-ratios method to account for differences in library size and RNA composition. A generalized linear model based on the negative binomial distribution was fitted, with group as the main explanatory variable; where relevant, batch, sequencing run, or other technical covariates were included in the design formula to control for potential confounders. Dispersion parameters were estimated using empirical Bayes shrinkage as implemented in DESeq2. Differentially expressed genes were defined using an adjusted *P*-value (Benjamini–Hochberg false discovery rate, FDR) threshold of < 0.05 and an absolute $\log_2$ fold change ($|\log_2 FC|) > 1$.

DNA methylation analysis

Whole-genome bisulfite sequencing (WGBS) data were processed following a standardized pipeline. Raw paired-end reads were first evaluated using FastQC (v0.12.1) to assess base quality, GC content, sequence duplication, and adapter contamination. Adapter trimming and quality filtering were then conducted with Trim Galore (v0.6.10), which internally uses Cutadapt (Martin, 2011) for adapter removal. Default settings were employed unless stated otherwise, including automatic detection and trimming of Illumina adapters, removal of low-quality bases (Phred score < 20), and discarding of reads shorter than 20–30 bp after trimming (the exact threshold is provided in the Supplementary Methods). Post-trimming QC was re-assessed with FastQC and summarized via MultiQC (v1.25.1) to ensure improved read quality and the removal of adapter sequences.

Trimmed reads were aligned to the bisulfite-converted reference genome (mouse: GRCm39; human: GRCh38) using Bismark (v0.24.2) (Krueger and Andrews, 2011) with Bowtie2 (v2.5.3) as the underlying aligner. Alignments were performed in paired-end mode, using appropriate options for directional or non-directional libraries as applicable. Parameters were tuned to balance sensitivity and specificity (e.g. limits on mismatches in the seed region). PCR duplicates were identified and removed using deduplicate_bismark to minimize amplification bias. Mapping efficiency, duplication rates, and bisulfite conversion efficiency—estimated from non-CpG (CHH/CHG) cytosines—were summarized as key QC metrics.

Cytosine methylation calls were generated using bismark_methylation_extractor, producing strand-specific methylation information for CpG, CHG, and CHH contexts. For downstream quantitative analyses, only CpG sites with a minimum coverage (e.g. ≥ 5×) in all samples for a given comparison were retained. The methylation level at each CpG was calculated as the fraction of methylated reads over the total reads covering that site.

Differentially methylated regions were identified using methylKit (v1.34) (Akalin *et al.*, 2012). Analyses were performed separately for mouse and human data, and for each relevant pairwise comparison. MethylKit was run with parameters optimized for WGBS, including a minimum number of CpGs per region, a minimum region length, and a minimum mean methylation difference (≥ 20%) between groups. Only regions with sufficient coverage across all samples and passing a multiple testing-corrected significance threshold ($q < 0.01$) were retained. Differentially methylated genes were subsequently defined by intersecting DMRs with gene bodies using standard genomic annotations, requiring that at least one DMR overlap a given feature with the specified methylation difference.

Functional enrichment

To interpret the potential biological relevance of differentially expressed genes (DEGs) and differentially methylated genes (DMGs), functional enrichment analyses were conducted using clusterProfiler (v4.12.0) (Yu *et al.*, 2012) within R (v4.4.0). Gene Ontology (GO) enrichment was performed with the enrichGO function. KEGG pathway enrichment was carried out using enrichKEGG, specifying the appropriate organism code (mouse: 'mmu'; human: 'hsa'). Where indicated, Reactome pathway enrichment was additionally evaluated using ReactomePA (v1.46.0) or the Reactome interface in clusterProfiler.

For overrepresentation analyses, the gene universe was carefully defined to minimize bias: for RNA-seq, the background consisted of all genes that passed expression and QC filters (i.e. testable by DESeq2); for WGBS, it consisted of all genes with sufficient CpG coverage to be included in DMR/DMG calling. Overrepresentation was tested using the hypergeometric distribution, as implemented in clusterProfiler, by comparing the observed overlap between the query gene set (e.g. DEGs or DMGs) and each functional category with the expected overlap under random sampling from the background. *P*-value were adjusted for multiple testing using the Benjamini–Hochberg procedure, and terms with adjusted *P*-value (FDR) and *q*-value < 0.01 were initially considered significantly enriched.

We performed Gene Set Enrichment Analysis (GSEA) using the gseGO and gseKEGG functions in clusterProfiler. For GSEA, all genes were ranked according to $\log_2$ fold change from DESeq2, and enrichment scores were computed for predefined GO and KEGG gene sets. Statistical significance was assessed by permutation testing, followed by Benjamini–Hochberg correction. In line with conventional GSEA practice, FDR < 0.25 was used as a discovery threshold, and pathways with FDR < 0.05 were highlighted as particularly robust. For selected comparisons, we also examined Reactome gene sets and, for methylation data, transcription factor target enrichment using web-based ChEA3.

References

Akalin A, Kormaksson M, Li S, Garrett-Bakelman FE, Figueroa ME, Melnick A, Mason CE. methylKit: a comprehensive R package for the analysis of genome-wide DNA methylation profiles. *Genome Biol* 2012;**13**:R87.

Kim D, Langmead B, Salzberg SL. HISAT: a fast spliced aligner with low memory requirements. *Nat Methods* 2015;**12**:357–360.

Krueger F, Andrews SR. Bismark: a flexible aligner and methylation caller for bisulfite-seq applications. *Bioinformatics* 2011;**27**:1571–1572.

Love MI, Huber W, Anders S. Moderated estimation of fold change and dispersion for RNA-seq data with DESeq2. *Genome Biol* 2014;**15**:550.

Martin M. Cutadapt removes adapter sequences from high-throughput sequencing reads. *EMBnet J* 2011;**17**:10–12.

Yu G, Wang L-G, Han Y, He Q-Y. clusterProfiler: an R package for comparing biological themes among gene clusters. *Omics* 2012;**16**:284–287.
