# Supplementary material for: Multi-omics and developmental comparison of direct and conventional warming methods in vitrified human and mouse cleavage-stage embryos
Source: Hum Reprod. 2026 Jul 6;41(9):1522–33. doi: 10.1093/humrep/deag105 (PMC13550675; doi:10.1093/humrep/deag105)
Supplement: Supplementary file 12 [file deag105_supplementary_table_s1.pdf]

Human Reproduction, 2026, 1–1
https://doi.org/10.1093/humrep/deag105
Supplementary Data

human reproduction
OXFORD

Supplementary Table S1. Reported simplified embryo warming protocols in published studies.

Study (author, year)	Publication type	Method	Kit/brand	Warming solution composition (CPA)	Embryo stage	Main outcomes
Jiang *et al.* (2024) (JARG)	Article	Single-step vs standard	FUJIFILM Irvine—Vit Kit Warm NX	Standard: TS 1.0 M trehalose (37°C, 1 min) → DS 0.5 M trehalose (4 min) → WS 0 M (8 min); Single-step: TS 1.0 M trehalose (1 min only)	Blastocyst	100% survival (n = 952); no diff. in live birth; ↑ multiple gestations
Fucci *et al.* (2025) (RBMO)	Article	Short (fast) vs long protocol	Vitrolife RapidWarm Omni™ kit	Short: Warm1 sucrose (1 min) → Warm4 no sucrose (1 min under oil); Long: Warm1 sucrose (1 min) → Warm2 sucrose (3 min) → Warm3 sucrose (5 min) → Warm4 no sucrose (5 min) (conc. not available)	Cleavage + blastocyst	Equal survival; ↓ miscarriage (18% vs 32%); ↑ ongoing pregnancy
Licata *et al.* (2025) (JARG)	Article	Diluted one-step vs standard	FUJIFILM Irvine—Vit Kit Warm NX	Diluted one-step: DS 0.5 M trehalose (37°C, 3 min); Standard: TS 1.0 M trehalose (1 min) → DS 0.5 M trehalose (3 min) → WS 0.5 M trehalose (5 min)	Blastocyst	94% vs 98% survival (ns); no diff. in re-expansion
Shioya *et al.* (2025) (Hum Reprod)	Article	One-step vs multi-step	FUJIFILM Irvine—Vit Kit Warm NX	Multi-step: TS 1.0 M trehalose (37°C, 1 min) → DS 0.5 M trehalose (4 min) → WS 0 M trehalose (8 min); One-step: TS 1.0 M trehalose (1 min only)	Cleavage + blastocyst	100% survival both stages; less collapse in one-step group
Lammers *et al.* (2025) (Reprod Sci)	Article	Ultra-fast vs standard	Vitrolife Rapid Warm Blast® kit	Ultra-fast: Warm1 0.25 M sucrose (2 min); Standard: Warm1 0.25 M sucrose (2 min) → Warm2 0.125 M sucrose (3 min) → Warm3 0 M Sucrose (10 min)	Blastocyst	100% survival; comparable re-expansion
Liebermann *et al.* (2024, 2026) (RBMO)	Article	One-step rehydration vs multi-step	Irvine—Vit Kit Warm (sucrose-based)	Multi-step: TS 1.0 M sucrose (1 min, 37°C) → DS 0.5 M sucrose (5 min) →WS 0 M sucrose (5 min); One-step: TS 1.0 M sucrose (1 min, 37°C)	Blastocyst	Equal survival; ↑ ongoing pregnancy and implantation; ↓ miscarriage; no diff. in live birth rates, gestational age and birth weight
Chan *et al.* (2025) (Fertil. Reprod.)	Article	Direct warming vs conventional	FUJIFILM Irvine—Vit Kit Warm NX Vitrolife, Embryo Culture, GTL	Multi-step: TS 1.0 M trehalose (37°C, 1 min) → DS 0.5 M trehalose (4 min) → WS 0 M trehalose (8 min); One-step: GTL no CPA (1 min only)	Blastocyst	Equal survival; no diff. in live birth;

This table summarizes simplified embryo warming methods (single-step, one-step, ultra-fast, and diluted one-step) compared with conventional multi-step protocols. Information includes publication type, commercial kit (when specified), cryoprotective agent (CPA) composition, embryo developmental stage, and reported outcomes. CPA, cryoprotective agent; TS, thawing solution; DS, dilution solution; WS, washing solution; RBMO, Reproductive BioMedicine Online; JARG, Journal of Assisted Reproduction and Genetics; Hum Reprod, Human Reproduction; Reprod Sci, Reproductive Science.
