# Supplementary material for: Multi-omics and developmental comparison of direct and conventional warming methods in vitrified human and mouse cleavage-stage embryos
Source: Hum Reprod. 2026 Jul 6;41(9):1522–33. doi: 10.1093/humrep/deag105 (PMC13550675; doi:10.1093/humrep/deag105)
Supplement: Supplementary file 13 [file deag105_supplementary_table_s2.pdf]

Human Reproduction, 2026, 1–1
https://doi.org/10.1093/humrep/deag105
Supplementary Data

human reproduction OXFORD

Supplementary Table S2. Number of transferred embryos, gestational sacs, and live-born pups in conventional and direct warming groups.

	Implantation		Live birth rate	
	**Conventional warming**	**Direct warming**	**Conventional warming**	**Direct warming**
FET	47	63	45	56
Number of gestational sacs	39	52	–	–
Number of pups	–	–	32	39
Rate	0.830	0.825	0.711	0.696
