# Supplementary material for: Multi-omics and developmental comparison of direct and conventional warming methods in vitrified human and mouse cleavage-stage embryos
Source: Hum Reprod. 2026 Jul 6;41(9):1522–33. doi: 10.1093/humrep/deag105 (PMC13550675; doi:10.1093/humrep/deag105)
Supplement: Supplementary file 14 [file deag105_supplementary_table_s3.pdf]

Human Reproduction, 2026, 1–1
https://doi.org/10.1093/humrep/deag105
Supplementary Data

human reproduction
OXFORD

Supplementary Table S3. Developmental milestones achieved by neonatal mice in conventional and direct warming groups.

Postnatal day (PND)	Developmental milestone	Conventional warming	Direct warming
PND 1	Hairless, closed eyes, normal body weight	Achieved	Achieved
PND 3–5	Skin pigmentation, visible hair follicles	Achieved	Achieved
PND 7	Fur on dorsal side, pinnae detached	Achieved	Achieved
PND 10	Eyes begin to open, improved movement	Achieved	Achieved
PND 14	Full eye opening, solid food intake starts	Achieved	Achieved
PND 18	Independent feeding behavior	Achieved	Achieved
PND 21 (Weaning)	Full weaning, normal body weight and length	Achieved	Achieved
