# Supplementary material for: Multi-omics and developmental comparison of direct and conventional warming methods in vitrified human and mouse cleavage-stage embryos
Source: Hum Reprod. 2026 Jul 6;41(9):1522–33. doi: 10.1093/humrep/deag105 (PMC13550675; doi:10.1093/humrep/deag105)
Supplement: Supplementary file 15 [file deag105_supplementary_table_s4.pdf]

Human Reproduction, 2026, 1–1
https://doi.org/10.1093/humrep/deag105
Supplementary Data

human reproduction

OXFORD

Supplementary Table S4. Morphological assessment of cleavage-stage embryos selected for parallel sequencing following conventional or direct warming.

ID	Embryos cell no. (freeze)	Embryos cell no. (warmed)	Further cleavage observed after thawing within 4 h (Y/N)	Groups
Emb1	8C	8C	Y	Conventional
Emb2	8C	8C	Y	Conventional
Emb3	7C	7C	Y	Conventional
Emb4	7C	7C	N	Conventional
Emb5	6C	6C	N	Conventional
Emb6	8C	8C	Y	Direct warm
Emb7	8C	8C	N	Direct warm
Emb8	8C	5C	Y	Direct warm
Emb9	7C	4C	N	Direct warm
Emb10	8C	8C	N	Direct warm

Summary of cell number before freezing and after thawing, and observation of further cleavage within 4 h post-thaw, in human cleavage-stage embryos subsequently used for parallel sequencing (scRNA-seq and DNA methylation profiling). Embryos were assigned to either the conventional warming (n = 5) or direct warming (n = 5) group. The absence of cleavage within 4 h does not preclude the possibility of subsequent division, as embryos were assessed within a fixed timeframe to permit molecular analysis. The timing of cleavage resumption may depend on the mitotic stage at which vitrification occurred.
