# Supplementary material for: Multi-omics and developmental comparison of direct and conventional warming methods in vitrified human and mouse cleavage-stage embryos
Source: Hum Reprod. 2026 Jul 6;41(9):1522–33. doi: 10.1093/humrep/deag105 (PMC13550675; doi:10.1093/humrep/deag105)
Supplement: Supplementary file 16 [file deag105_supplementary_table_s7.pdf]

Human Reproduction, 2026, 1–1
https://doi.org/10.1093/humrep/deag105
Supplementary Data

human reproduction
OXFORD

Supplementary Table S7. Functional enrichment analysis of differentially expressed genes (DEGs) across methods in human and mice embryos.

Methods	Human C1 vs C2	Mice C1 vs C2	Mice FE vs C1	Mice FE vs C2
GO BP	n/a	n/a	n/a	UP: Negative regulation of BMP signaling pathway<br>DOWN: Positive regulation of dephosphorylation; positive regulation of protein dephosphorylation; regulation of protein dephosphorylation
GO MF	n/a	n/a	n/a	UP: GTPase activity; GDP binding<br>DOWN: n/a
GO CC	n/a	UP: female pronucleus<br>DOWN: n/a	n/a	n/a
KEGG	UP: n/a<br>DOWN: cytoskeleton in muscle cells; arrhythmogenic right ventricular cardiomyopathy	n/a	UP: breast cancer; Gastric cancer<br>DOWN: n/a	UP: colorectal cancer<br>DOWN: n/a
GSEA	n/a	n/a	n/a	n/a
Reactome	n/a	n/a	n/a	n/a

Gene ontology (GO) enrichment for biological process (BP), molecular function (MF), and cellular component (CC), along with KEGG pathway, GSEA, and Reactome analyses are shown. UP: pathways enriched in upregulated genes; DOWN: pathways enriched in downregulated genes; n/a: no significant enrichment detected (q-value < 0.01). Differentially expressed genes (DEGs).

Reference for the cutoff (q-value < 0.01):

Anisimova AS, Kolyupanova NM, Makarova NE, Egorov AA, Kulakovskiy IV, Dmitriev SE. Human tissues exhibit diverse composition of translation machinery. *Int J Mol Sci* 2023;**24**:8361.
Bhate A, Parker DJ, Bebee TW, Ahn J, Arif W, Rashan EH, Chorghade S, Chau A, Lee J-H, Anakk S, *et al.* ESRP2 controls an adult splicing programme in hepatocytes to support postnatal liver maturation. *Nat Commun* 2015;**6**:8768.
Huang L, Qin S, Xia L, Ma F, Chen L. Integrative single-cell sequencing analysis distinguishes survival-associated cells from the breast cancer microenvironment. *Cancer Med* 2023;**12**:12896–12911.
Solé C, Moliné T, Vidal M, Ordi-Ros J, Cortés-Hernández J. An exosomal urinary miRNA signature for early diagnosis of renal fibrosis in lupus nephritis. *Cells* 2019;**8**:773.
Wang H, Wang Y, Yang J, Zhao Q, Tang N, Chen C, Li H, Cheng C, Xie M, Yang Y, *et al.* Tissue- and stage-specific landscape of the mouse translatome. *Nucleic Acids Res* 2021;**49**:6165–6180.
Wu Y, Yang L, Zhang L, Zheng X, Xu H, Wang K, Weng X. Identification of a four-gene signature associated with the prognosis prediction of lung adenocarcinoma based on integrated bioinformatics analysis. *Genes (Basel)* 2022;**13**:238.
