# Supplementary material for: Multi-omics and developmental comparison of direct and conventional warming methods in vitrified human and mouse cleavage-stage embryos
Source: Hum Reprod. 2026 Jul 6;41(9):1522–33. doi: 10.1093/humrep/deag105 (PMC13550675; doi:10.1093/humrep/deag105)
Supplement: Supplementary file 17 [file deag105_supplementary_table_s8.pdf]

Human Reproduction, 2026, 1–1
https://doi.org/10.1093/humrep/deag105
Supplementary Data

human reproduction

OXFORD

Supplementary Table S8. Functional enrichment analysis of DMGs/DMRs across methods in human and mice embryos.

Methods	Human C1 vs C2	Mice C1 vs C2
GO BP/MF/CC	n/a	n/a
ChEA3	n/a	n/a
KEGG	n/a	n/a
GSEA	n/a	n/a
Reactome	n/a	n/a

Gene ontology (GO) enrichment for biological process (BP), molecular function (MF), and cellular component (CC), along with ChEA3, KEGG pathway, GSEA, and Reactome analyses are shown (q-value < 0.01). Differentially methylated regions (DMRs); differentially methylated genes (DMGs).

Reference for the cutoff (q-value < 0.01):

Anisimova AS, Kolyupanova NM, Makarova NE, Egorov AA, Kulakovskiy IV, Dmitriev SE. Human tissues exhibit diverse composition of translation machinery. *Int J Mol Sci* 2023;**24**:8361.
Bhate A, Parker DJ, Bebee TW, Ahn J, Arif W, Rashan EH, Chorghade S, Chau A, Lee J-H, Anakk S, *et al.* ESRP2 controls an adult splicing programme in hepatocytes to support postnatal liver maturation. *Nat Commun* 2015;**6**:8768.
Huang L, Qin S, Xia L, Ma F, Chen L. Integrative single-cell sequencing analysis distinguishes survival-associated cells from the breast cancer microenvironment. *Cancer Med* 2023;**12**:12896–12911.
Solé C, Moliné T, Vidal M, Ordi-Ros J, Cortés-Hernández J. An exosomal urinary miRNA signature for early diagnosis of renal fibrosis in lupus nephritis. *Cells* 2019;**8**:773.
Wang H, Wang Y, Yang J, Zhao Q, Tang N, Chen C, Li H, Cheng C, Xie M, Yang Y, *et al.* Tissue- and stage-specific landscape of the mouse translatome. *Nucleic Acids Res* 2021;**49**:6165–6180.
Wu Y, Yang L, Zhang L, Zheng X, Xu H, Wang K, Weng X. Identification of a four-gene signature associated with the prognosis prediction of lung adenocarcinoma based on integrated bioinformatics analysis. *Genes (Basel)* 2022;**13**:238.

© The Author(s) 2026. Published by Oxford University Press on behalf of European Society of Human Reproduction and Embryology.
This is an Open Access article distributed under the terms of the Creative Commons Attribution License (https://creativecommons.org/licenses/by/4.0/), which permits unrestricted reuse, distribution, and reproduction in any medium, provided the original work is properly cited.
Human Reproduction, 2026, 00, 1–30
https://doi.org/10.1093/humrep/deag105
Original Article
